# Immune Checkpoint Molecules—Inherited Variations as Markers for Cancer Risk

**DOI:** 10.3389/fimmu.2020.606721

**Published:** 2021-01-14

**Authors:** Marta Wagner, Monika Jasek, Lidia Karabon

**Affiliations:** Laboratory of Genetics and Epigenetics of Human Diseases, Department of Experimental Therapy, Hirszfeld Institute of Immunology and Experimental Therapy, Polish Academy of Sciences, Wroclaw, Poland

**Keywords:** immune checkpoint molecules, CTLA-4, PD-1/PD-L1, BTLA, TIM-3, LAG-3, polymorphisms, cancer risk

## Abstract

In recent years, immunotherapy has been revolutionized by a new approach that works by blocking receptors called immune checkpoints (IC). These molecules play a key role in maintaining immune homeostasis, mainly by suppressing the immune response and by preventing its overactivation. Since inhibition of the immune response by IC can be used by cancer to avoid recognition and destruction by immune system, blocking them enhances the anti-tumor response. This therapeutic approach has brought spectacular clinical effects. The ICs present heterogeneous expression patterns on immune cells, which may affect the effectiveness of immunotherapy. The inherited genetic variants in regulatory regions of ICs genes can be considered as potential factors responsible for observed inter-individual differences in ICs expression levels on immune cells. Additionally, polymorphism located in exons may introduce changes to ICs amino acid sequences with potential impact on functional properties of these molecules. Since genetic variants may affect both expression and structure of ICs, they are considered as risk factors of cancer development. Inherited genetic markers such as SNPs may also be useful in stratification patients into groups which will benefit from particular immunotherapy. In this review, we have comprehensively summarized the current understanding of the relationship between inherited variations of *CTLA-4, PDCD1*, *PD-L1, BTLA*, *TIM-3*, and *LAG-3* genes in order to select SNPs which can be used as predictive biomarkers in personalized evaluation of cancer risk development and outcomes as well as possible response to immunotherapy.

## Introduction

Immune checkpoints (ICs) are key receptors that inhibit the immune response and prevent from its overactivation. Under normal conditions, this mechanism is responsible for maintaining tolerance to its own antigens; however; it can be used by cancer cells to avoid recognition and destruction (1). A discovery made by prof. Allison and prof. Honjo (2, 3) (Nobel Prize in 2018), indicating that blocking these molecules elicits an anti-cancer response, has opened up new perspectives for cancer treatment.

Recently, a number of receptors belonging to the immune checkpoint family have been discovered. However, only two of them CTLA-4 and PD-1 are primarily and broadly explored and their blockade is used as a therapeutic procedure in the routine treatment of several cancers: among others, in the treatment of advanced melanoma, non-small cell lung cancer, non-Hodgkin’s lymphoma, and kidney cancer (4).

The unique benefits of IC blocking include: efficacy against a broad panel of tumors, extended survival, and long-term healing effect after treatment (even over 10 years).

In spite of the evidence of considerable clinical relevance of anti-CTLA-4 and anti-PD-1 antibodies there is still a need for deeper understanding of the relation between the host’s genetics and mechanisms involved in the regulation of these pathways; especially when considering that only some patients (20–40%) respond to therapy, while a small proportion of patients experience rapid progression or an increased risk of death (5). In addition, some patients develop severe, sometimes fatal, adverse effects associated with the autoimmune response (5).

Therefore, in recent years new inhibitory pathways have started to be intensively studied as promising targets for immune checkpoint blocking therapy. Several potential molecules as well as their ligands are considered (4), among them: the B and T lymphocyte attenuator (BTLA), the Lymphocyte activation gene 3 (LAG-3), the T cell Immunoglobulin 3 (TIM-3) and the T cell immunoglobulin and ITIM domain (TIGIT) (4).

The immune status of cancer is modulated by many factors including patients’ immune reactivity which may be affected by single nucleotide polymorphisms (SNPs) of immune related genes (6). These SNPs may occur in regulatory regions and cause changes leading to damaging or introducing binding sites for transcription factors (TFs) or miRs, and in that way exert the influence on the expression level of encoded molecules as well as affect chromatin accessibility or DNA-looping (7). They may also introduce changes to protein structure which may affect function of these molecules. Hence, genetic polymorphism may impair function of molecules important for effective activity of anti-tumor response, such as CTLA-4; the SNPs of *CTLA-4* and other immune checkpoints molecules have been studied in the context of variety types of cancers.

This review will summarize current knowledge about the impact of the genetic variants of *CTLA-4*, *PDCD1*, *PD-L1, BTLA, TIM-3*, and *LAG-3* on cancer risk. In case of the *CTLA-4*, *PDCD1* as well as *PD-L1* genes, a considerable number of meta-analyses combining the results of numerous studies are available in the literature and in the next paragraphs their results will be preferentially analyzed. In the case of other IC molecules, the results of individual studies in particular cancers will be presented. Of note, we omitted those reports that analyzed only a small number of individuals in case-control groups.

## Cytotoxic T Lymphocyte-Associated Antigen-4

**Figure 1:**
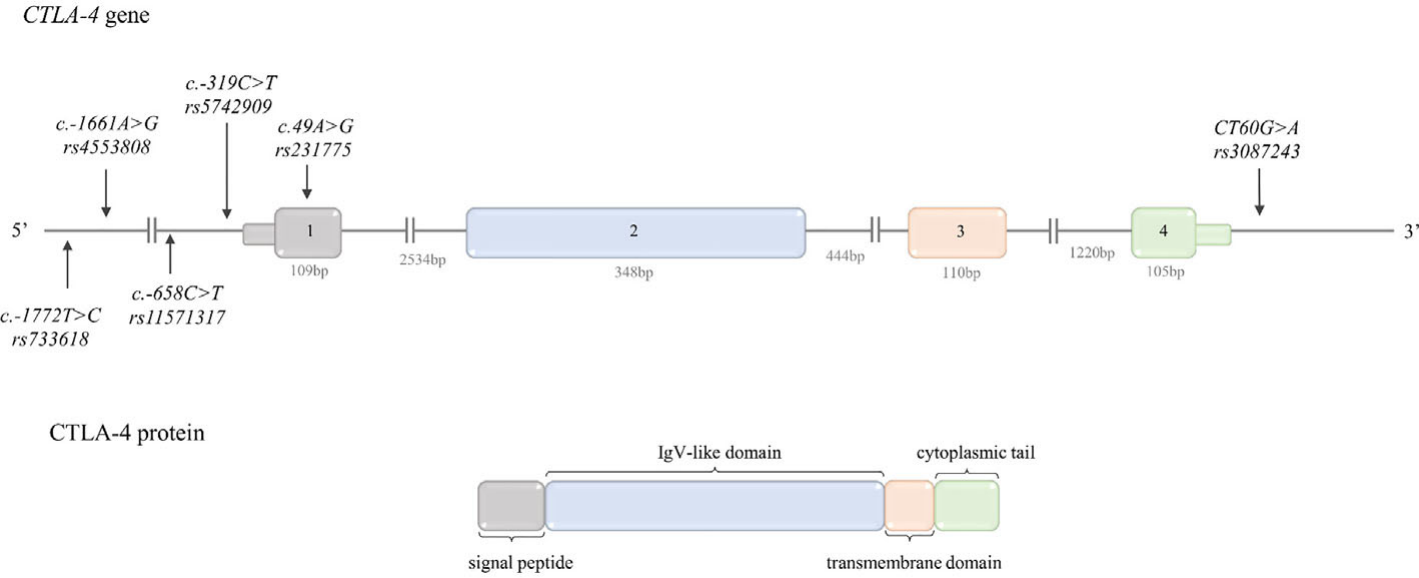
Schematic CTLA-4 gene and protein structure. Top: *CTLA-4* gene structure. The figure shows the polymorphisms described in the review and the lengths of the exons and introns. Bottom: CTLA-4 protein structure. The colors indicate which region of the protein is encoded by which exon.

The cytotoxic T lymphocyte-associated antigen-4 (CTLA-4; CD152) was discovered in 1987 by Brunet and colleagues (8). CTLA-4 is an inhibitory cell surface receptor belonging to the immunoglobulin-like receptor superfamily, structurally similar to CD28 (9). It is comprised of four domains, including a signal peptide, an extracellular ligand-binding domain, a transmembrane domain, and a short cytoplasmic tail (**Figure 1**) (8). CTLA-4 forms a covalently linked heterodimer that binds to the same ligands as CD28 - CD80 (B7.1) and CD86 (B7.2) (10–12), although with significantly higher affinity and avidity (12). CTLA-4 is expressed by conventional effector T cells (T_eff_) upon activation, and constitutively by the regulatory T cells (T_regs_) (13). Although expression of CTLA-4 is primarily restricted to T cells, its presence on B cells and other cell types has been also described [reviewed in (13–15)]. The expression of CTLA-4 on different types of tumor cells has been also demonstrated (16, 17).

CTLA-4 engagement, upon TCR activation, decreases T cells response through the inhibition of co-stimulatory signals coming from CD28. This molecule is vital for regulation of T-cell homeostasis and self-tolerance (18).

From an “oncological” point of view, the most important feature of CTLA-4 is its engagement in evading immune surveillance by cancer cells, since cancer cells may employ mechanisms leading to upregulation of CTLA-4 and as consequence repression of immune response toward tumor cells (18). The discovery made by Prof. Allison’s group showing in murine model of tumor that the blocking of CTLA-4 caused an enhanced antitumor immunity (3), gave a new perspective on therapeutic approaches to cancer.

After more than decade later the idea of Prof. Allison’s team achieved an enormous success, when it was proved that antibody against CTLA-4 – ipilimumab, was active in patients with metastatic melanoma. Moreover, a long-term response to this treatment was observed in patients with prolonged stable disease (19). Finally, in 2011 FDA approved ipilimumab for treatment in advanced melanoma.

The human CTLA-4 exists in two main isoforms: the full length CTLA-4 (flCTLA-4) and soluble CTLA-4 (sCTLA-4), which is devoid of the transmembrane domain (20). The flCTLA-4 is responsible for inhibition of T cell activation which leads to T-cell anergy. The sCTLA-4 by antagonizing this process maintains the immune response in balance (21). Some of genetic polymorphisms of *CTLA-4* gene may have impact on the ratio between these two isoforms and in consequence on T cell activity (22, 23).

The *CTLA-4* gene is located between the *CD28* and *ICOS* genes on chromosome 2q33.2. The structure of *CTLA-4* gene is presented in **Figure 1**.

The transcriptional regulation of *CTLA-4* gene expression is not fully understood. It is regulated by the NFAT TF, since it has been shown that NFAT binds to the *CTLA-4* promoter region (24) and CTLA-4 expression directly correlated with NFAT level. Also, FoxP3 is a direct activator of the *CTLA-4* gene (25). In addition, GATA3 binds to the *CTLA-4* proximal promoter in bortezomib-treated CD4^+^ T cells, and GATA3 expression can enhance *CTLA-4* promoter activity in a dose-dependent manner (26). Recently, was shown that Zfp281 TF belonging to the zing finger family, negatively regulated CTLA-4 expression by direct binding to GC rich sites in its promoter (27). The expression of CTLA-4 is also regulated by different miRNAs. The regulations of ICs by miRs have been recently described in the review by Omar et al. (28).

### *CTLA-4* Gene Polymorphisms

As mentioned earlier, the upregulation of CTLA-4 expression is one of the mechanisms adopted by tumor cells to evade anti-tumor immune response. These mechanisms may be modulated by inherited genetic variants. Below we described the most intensively examined genetic variants of *CTLA-4* in human tumors together with their functional relevance, if such data were available in literature. The frequency of the *CTLA-4* genetic variants described below in different populations is presented in **Table 1**.

**Table 1:** Frequency of genetic variants of *CTLA-4*, *PDCD1*, *PD-L1*, *BTLA*, *HAVCR2*, and *LAG3* genes (in different human populations) for which the association with cancer risk has been investigated.

GENE	GENETIC VARIANT		FREQUENCY IN HUMAN POPULATIONS (1000Genomes Study)			
	Reference SNP (rs)	Common Name	Global	European	South Asian	East Asian
*CTLA-4* (+ strand)	rs733618 T>C	CTLA-4c.-1722 T>C	T=0.83 C=0.17	T=0.94 C=0.06	T=0.87 C=0.13	T=0.61 C=0.39
	rs4553808 A>G	CTLA-4c.-1661A>G	A=0.87 G=0.13	A=0.83 G=0.17	A=0.94 G=0.06	A=0.90 G=0.10
	rs11571317 C>T	CTLA-4c.-658 C>T	C=0.98 T=0.02	C=0.92 T=0.08	C=0.99 T=0.01	C=1.00 T=0.00
	rs5742909 C>T	CTLA-4c.-319C>T	C=0.95 T=0.05	C=0.92 T=0.08	C=0.97 T=0.03	C=0.90 T=0.10
	rs231775 A>G	CTLA-4c.49A>G	A=0.57 G=0.43	A=0.64 G=0.36	A=0.69 G=0.31	A=0.36 G=0.64
	rs3087243 G>A	CTLA-4CT60	G=0.63 A=0.37	G=0.53 A=0.47	G=0.37 A=0.63	G=0.74 A=0.26
*PDCD1* *PD1* (- strand)	rs36084323 C>T G>A^*^	PD-1.1 G>A	C=0.85 T=0.15	C=0.99 T=0.01	C=0.94 T=0.06	C=0.53 T=0.47
	rs11568821 C>T G>A^*^	PD-1.3 G>A	C=0.96 T=0.04	C=0.88 T=0.12	C=0.97 T=0.03	C=1.00 T=0.00
	rs2227981 G>A C>T^*^	PD-1.5 C>T	G=0.65 A=0.35	G=0.60 A=0.40	G=0.76 A=0.24	G=0.73 A=0.27
	rs10204525 C>T A>G^*^	PD-1.6 A>G	C=0.65 T=0.35	C=0.88 T=0.12	C=0.81 T=0.19	C=0.34 T=0.66
	rs2227982 G>A C>T^*^	PD-1.9 C>T	G=0.86 A=0.14	G=0.99 A=0.01	G=0.94 A=0.06	G=0.55 A=0.45
	rs7421861 A>G T>C^*^		A=0.73 G=0.27	A=0.65 G=0.35	A=0.62 G=0.38	A=0.83 G=0.17
	rs41386349 G>A C>T^*^		G=0.85 A=0.15	G=0.90 A=0.10	G=0.86 A=0.14	G=0.78 A=0.22
*PD-L1* (+ strand)	rs4143815 G>C		G=0.72 C=0.28	G=0.67 C=0.33	G=0.80 C=0.20	G=0.43 C=0.57
	rs2297136 A>G		A=0.67 G=0.33	A=0.55 G=0.45	A=0.67 G=0.33	A=0.81 G=0.19
	rs10815225 G>C		G=0.84 C=0.16	G=0.87 C=0.13	G=0.82 C=0.18	G=0.92 C=0.08
	rs4742098 A>G		A=0.71 G=0.29	A=0.70 G=0.30	A=0.81 G=0.19	A=0.46 G=0.54
	rs17718883 C>G		C=0.99 G=0.01	C=0.99 G=0.01	C=1.00 G=0.00	C=1.00 G=0.00
	rs2890657 G>C		G=0.79 C=0.21	G=0.77 C=0.23	G=0.87 C=0.13	G=0.50 C=0.50
	rs2890658 C>A		C=0.84 A=0.16	C=0.90 A=0.10	C=0.82 A=0.18	C=0.85 A=0.15
	rs822338 T>C		T=0.72 C=0.28	T=0.71 C=0.29	T=0.76 C=0.24	T=0.45 C=0.55
*BTLA* (- strand)	rs2705511 A>C T>G^*^		A=0.55 C=0.45	A=0.76 C=0.24	A=0.42 C=0.58	A=0.28 C=0.72
	rs1982809 A>G T>C^*^		A=0.57 G=0.43	A=0.77 G=0.23	A=0.59 G=0.41	A=0.21 G=0.79
	rs9288952 A>G T>C^*^	BTLAc.800 G>A	A=0.65 G=0.35	A=0.96 G=0.04	A=0.80 G=0.20	A=0.72 G=0.28
	rs76844316 T>G A>C^*^	BTLAc.590 A>C	T=0.96 G=0.04	T=1.00 G=0.00	T=0.99 G=0.01	T=0.94 G=0.06
	rs16859633 T>C A>G^*^		T=1.00 C=0.00	T=1.00 C=0.00	T=1.00 C=0.00	T=1.00 C=0.00
	rs9288953 C>T G>A^*^		C=0.73 T=0.27	C=0.68 T=0.32	C=0.74 T=0.26	C=0.46 T=0.54
	rs2705535 C>T G>A^*^		C=0.87 T=0.13	C=0.98 T=0.02	C=0.96 T=0.04	C=0.80 T=0.20
	rs1844089 G>A C>T^*^		G=0.70 A=0.30	G=0.93 A=0.07	G=0.81 A=0.19	G=0.75 A=0.25
	rs2705565 C>T G>A^*^		C=0.78 T=0.22	C=0.96 T=0.04	C=0.88 T=0.12	C=0.80 T=0.20
	rs2633580 C>G G>C^*^		C=0.70 G=0.30	C=0.93 G=0.07	C=0.81 G=0.19	C=0.75 G=0.25
	rs2931761 T>G A>C^*^		T=0.99 G=0.01	T=1.00 G=0.00	T=1.00 G=0.00	T=0.99 G=0.01
	rs2633562 T>C A>G^*^		T=0.80 C=0.20	T=0.96 C=0.04	T=0.88 C=0.12	T=0.80 C=0.20
	rs16859629 T>C A>G^*^		T=0.96 C=0.04	T=1.00 C=0.00	T=0.99 C=0.01	T=0.94 C=0.06
	rs2171513 G>A C>T^*^		G=0.68 A=0.32	G=0.96 A=0.04	G=0.80 A=0.20	G=0.78 A=0.22
	rs312270 T>C A>G^*^		T=0.77 C=0.23	T=0.67 C=0.33	T=0.63 C=0.37	T=0.80 C=0.20
*HAVCR2* *TIM3* (- strand)	rs891246256 G>A C>T^*^	-1541 C>T	G=1.00 A=0.00	G=1.00 A=0.00	G=1.00 A=0.00	G=1.00 A=0.00
	rs10053538 C>A G>T^*^	-1516 G>T	C=0.93 A=0.07	C=0.95 A=0.05	C=0.88 A=0.12	C=0.94 A=0.06
	rs4704853 G>A C>T^*^	-822 C>T	G=0.85 A=0.15	G=0.81 A=0.19	G=0.96 A=0.04	G=0.98 A=0.02
	rs10515746 C>A G>T^*^	-574G>T	C=0.85 A=0.15	C=0.81 A=0.19	C=0.96 A=0.04	C=0.98 A=0.02
	rs1036199 A>C T>G^*^	+4259T>G	A=0.88 C=0.12	A=0.82 C=0.18	A=0.97 C=0.03	A=0.98 C=0.02
	rs4704846 A>G T>C^*^		A=0.84 G=0.16	A=0.81 G=0.19	A=0.97 G=0.03	A=0.98 G=0.02
*LAG3* (+ strand)	rs2365094 G>C		G=0.68 C=0.32	G=0.73 C=0.27	G=0.63 C=0.37	G=0.55 C=0.45
	rs3782735 A>G		A=0.66 G=0.34	A=0.60 G=0.40	A=0.64 G=0.36	A=0.60 G=0.40

^*Reference SNPs are showed on forward orientation, if a gene is located on minus strand a reverse orientation* is also presented for reference SNPs. The Table was prepared based on dbSNP database.^

***rs4553808* (*CTLA-4c.-1661A>G*)** and ***rs733618 (CTLA-4c.-1722T>C*)** are located in the upstream regulatory region and may impact the transcription-associated binding activity of CCAAT-enhancer binding protein C/EBPβ and nuclear factor 1 (NF-1) TFs, respectively. The *in silico* analysis suggested that the change of adenine to guanine at -1661 position may lead to creation of a new binding site for a C/EBPβ TF (29, 30). The C/EBP family of TFs plays an important role in the regulation of many essential processes like cell cycle, hematopoiesis, and host immune response (31), the C/EBPβ is implicated in cell proliferation, apoptosis and transformation (30). Therefore, it can be anticipated that an extra C/EBPβ binding motif created by *CTLA-4c.-1661A>G* may increase the transcriptional activity and lead to higher expression of CTLA-4. Such a phenomenon was recently described for the rs975484*C>G* located in a protein arginine methyltransferase 1 gene (*PRMTI*) (32). This assumption has to be experimentally validated, however, if it appears to be correct, the CTLA-4c.-1661*G allele may be considered as potential cancer risk factor.

In contrast, the substitution of thymine to cytosine at position *-1722* was predicted to destroy a binding site for a NF-1 TF. It has been suggested that the NF-1 TF and element surrounding *-1772* position may regulate *CTLA-4* alternative splicing, since CTLA-4c.-1722*C allele was associated with higher expression of sCTLA-4 (29). Hence, sCTLA-4 antagonize anergy of T cells, the CTLA-4c.-1722*C allele may be considered as protective allele in terms of developing cancer, and opposite effect should be observed for CTLA-4c.-1722*T allele (higher risk).

***rs11571317C>T* (*CTLA-4c.-658C>T*)** is located in 5’ UTR region. *In silico* analysis revealed that this polymorphism is located in potential binding site for a SP1 TF (*C* allele), and that presence of thymine at this site may lead to disruption of the SP1 binding motif. This observation suggests that CTLA-4c.-658*T allele may be associated with lower expression of CTLA-4 molecule (33). Hence, in the context of cancer, CTLA-4c.-658*C allele (higher expression of CTLA-4 molecule) may confer increased risk of cancer development.

***rs5742909*C>T (*CTLA-4c.-319C>T*)** is located in the promoter region. It has been demonstrated that this variant modified a binding site for a LEF1 TF. The presence of cytosine or thymine at position *-319* has been shown to have an impact on regulatory potential of the LEF1, which led to differential activation of the -*319C>T* variant by LEF1. More precisely, it has been demonstrated in the reporter assay that in the presence of LEF1, the activity of the reporter gene was higher for thymine at *-319* position in activated Jurkat cells (34). The CTLA-4c.-319*T allele has also been associated with significantly increased expression of *CTLA-4* mRNA and surface CTLA-4 expression on unstimulated peripheral blood mononuclear cells (PMBC) and CD4^+^ T cells, but not on CD8^+^ T cells (35, 36). Based on that, it may be assumed that CTLA-4c.-319*T allele is associated with higher expression of CTLA-4, and therefore, may increase a risk of cancer development and progression. Xiong and colleagues (37) investigated functional relevance of *CTLA-4c.-319C>T* and found that upon stimulation PBMCs derived from subjects with *CTLA-4c.-319 T/T* genotype had significantly lower proliferation ability, produced lower levels of IL-2 and IL-4, but on the contrary higher levels of TGF-β compared with PBMCs derived from subjects with *CTLA-4c.-319 C/T* or *CTLA-4c.-319 C/C* genotypes. Furthermore, the authors demonstrated, that stimulated PBMCs with *CTLA-4c.-319 T/T* genotype were significantly less cytotoxic towards CaSki cell line cells (HPV 16-positive cervical cancer cell line) than PBMCs from individuals with *CTLA-4c.-319 C/T* or *C/C* genotypes. The authors concluded, that the carriers of CTLA-4c.-319*T allele “have stronger negative regulation of T-cell proliferation and function, which might be the underlying mechanisms conferring cervical cancer susceptibility” (37).

***rs231775A>G* (*CTLA-4c.49A>G*)** is a non-synonymous SNP which leads to an amino acid change from threonine to alanine in position 17 of CTLA-4 leader peptide (Thr17Ala). The presence of the *CTLA-4c.49 A/A* (Thr17) genotype, as opposed to the *G/G* genotype (Ala17), was associated with significantly lower levels of T cells activation and lower proliferation of these cells. It has been also demonstrated that CTLA-4 molecule with (Thr17) had a higher capacity to bind CD80 and a stronger inhibitory effect on T cell activation compared to (Ala17) (38). Moreover, it was presumed that (Ala17) homozygotes may express one-third less CTLA-4 on the surface of T-cells than (Thr17) homozygotes (38). It was also postulated that the *CTLA-4c.49A>G* in the leader sequence alters the inhibitory function of CTLA-4 by influencing the rate of endocytosis or surface trafficking (35, 39). Taking into consideration the aforementioned observation it is reasonable to consider the CTLA-4c.49*A allele as a risk factor of cancer development.

***rs3087243G>A* (*CTLA-4CT60G>A)*** is situated in the 3’ UTR. The *CTLA-4CT60 G/G* genotype has been shown to be associated with lower expression of *sCTLA-4* transcript (23). Xiong and colleagues (37) in work described above investigated also functional relevance of *CTLA-4CT60G>*A SNP and observed the same functional effects for the CTLA-4CT60*G allele as for CTLA-4c.-319*T allele (37). On the other hand, the *CTLA-4CT60 A/A* genotype was associated with the approximately 40% increase in T_reg_ frequency in the peripheral blood (PB) of healthy donors (40). Based on data obtained from results mentioned above it can be inferred that the CTLA-4CT60*G allele constitutes cancer risk factor. The possible functional relevance of *CTLA-4* SNPs was summarized in **Table 2**.

**Table 2:** The possible functional relevance of polymorphisms in genes encoding immune checkpoint molecules.

GENE	Single nucleotide polymorphisms	Higher risk allele	Potential functional relevance and implication for cancer	Experimentally confirmed implication for cancer
***CTLA-4***	*rs733618T>C* *(CTLA-4c.-1722T>C)*	*T*	NF-1 TF binding site (29) allele C may destroy this motif;	allele C **↑** expression of sCTLA-4 (29)
	*rs4553808 A>G* *(CTLA-4c.-1661A>G)*	*G*	extra C/EBPβ TF binding site (29) ↑ CTLA-4 expression	
	*rs11571317C>T* *(CTLA-4c.-658C>T)*	*C*	SP1 TF binding site (33) allele T may destroy this motif (predicted ↓ CTLA-4 expression for T allele)	
	*rs5742909 C>T* *(CTLA-4c.-319C>T)*	*T*		-319*T: **↑** transcriptional activity in presence of LEF1 TF (34) **↑** mRNA and surface expression of *CTLA-4* in PBMCs (35, 36) *-319 T/T* ↓ cytotoxic for CaSki cells; proliferation; IL-2 and IL-4, **↑** TGF-β (37)
	*rs231775A>G* *(CTLA-4c.49A>G)*	*A*		*49 A/A* ↓ T cells activation and proliferation (38)
	*rs3087243G>A* *(CTLA-4CT60G>A)*	*G*		CTLA-4CT60*G ↑ mRNA and surface expression of *CTLA-4* in PBMCs (35, 36) *CT60G/G* ↓ sCTLA-4 mRNA ↓ (23) cytotoxic for CaSki cells; Proliferation; IL-2 and IL-4 ↑ TGF-β (37)
	CTLA-4g.*642AT (8_33)		In strong LD with *rs3087243G>A (CTLA-4CT60G>A)*	
***PDCD1***	*rs36084323C>T* *(PD-1.1 G>A REV)*	*G*	UCE-2 transcription regulators binding sites	PD-1.1*G ↑ promoter activity (41)
	*rs7421861 T>C*		may induce aberrant splicing, and lead to translational suppression (42)	
	*rs11568821C>T* *(PD-1.3 G>A)*	*A*	PD-1.3*A - disruption of RUNX1 TF binding site (43); aberrant PD-1 expression and deregulated lymphocyte activity (44)	
	*rs41386349 C>T*	*T*	putative enhancer-like region (45)	rs41386349* T allele - creation of negative cis-element for transcription; ↓ transcriptional activity of PD-1 in human T cells (45)
	*rs2227982 G>A* *(PD-1.9 C>T)*	*T*	nonsynonymous SNP in extracellular domain of PD-1 possibly altering the function of PD-1 (42, 44)	
	*rs2227981G>A* *(PD-1.5 C>T)*	*T*	synonymous variation (Ala268Ala) located in exon 5 possibly in LD with another polymorphism, which may alter PD-1 expression (44)	↑ % PD-1^+^CD4^+^ T cells in individuals with *PD-1.5 C/T* and *PD-1.5 T/T* (46)
	*rs10204525C>T* *(PD-1.6 G>A)*	*A*	located in 3’UTR in putative miRNA binding site	↓ PD-1 expression and ↑TNF-α, ↑ IFN-γ by miR-4717 in lymphocytes of chronic HBV patients with PD-1.6 *G/G* genotype (47) ↓ PD-1 expression in HBV patients with PD-1.6 *G/G* genotype (48)
***PD-L1***	*rs10815225G>C*	*G*	SP1 consensus motif	↑ *PD-L1* mRNA in gastric cancer patients with *rs10815225 G/G* genotype as compared to patients with the *rs10815225 G/C* genotype (49)
	*rs866066C>T*		potential TFs binding site	
	*rs2890657G>C*	*C*	located in intron 1, predicted to affect the c-Myb TF binding site	
	*rs17718883C>G*		missense variant causing the substitution from Pro to Arg, located in exon 4 (50)	
	*rs2890658C>A*		no data available	no data available
	*rs2297136A>G*	*A*	potential binding site for miR-296-5p (51)	expression in constructs containing rs2297136*G allele was significantly inhibited by miR-296-5p (51)
	*rs4143815G>C*	*G*	predicted putative binding site for miR-7-1*, miR-495, miR-298 (51) and miR-570 (51, 52)	expression in constructs containing rs4143815*C allele was significantly suppressed by miR-570 (52)
	*rs4742098A>G*	*G*	miR binding site	expression in constructs containing rs4742098*A allele was significantly suppressed by miR-138 (51)
	*rs822338T>C*		located in intron 1, predicted to affect the binding site for TAF1 and p300 (53)	
***BTLA***	*rs2633580C>G*		2KB Upstream Variant	
	*rs2705565C>T*		2KB Upstream Variant	
	*rs1844089G>A*		Intron Variant	
	*rs2705535 C>T*		Postulated potential role in splicing (54)	
	*rs9288953 C>T*	*T*	Predicted to activate six new splice sites in splicing enhancer motifs and break one splicing sites in silencer motifs ↑ potential BTLA expression (54)	
	*rs16859633T>C*		Missense Variant exon 2	
	*rs2633562T>C*		Intron Variant	
	*rs16859629T>C*		Intron Variant	
	*rs2931761G>T*		Missense Variant exon 3	
	*BTLAc.590A>C (rs76844316T>G)*	*A*	Missense Variant exon 4	BTLAc.590*A allele was associated with decreased inhibitory activity of BTLA (55)
	*BTLAc.800A>G* *(rs9288953C>T)*		Missense Variant exon 5	
	*rs2171513A>G*		3 Prime UTR Variant	
	*rs1982809T>C*	*C*		↓ mRNA expression in T cells of the CLL patients (56)
	*rs2705511A>C*		intragenic region between genes encoding CD200 and BTLA (-97820bp ||-3334bp	
***HAVCR2***	*rs891246256G>A* *(-1541C>T)*		2kb upstream SNPs	
	*rs10053538C>A* *(-1516G>T)*	*T*	putative binding site of p300 TF (57)	The presence of -1516*T allele associated with higher expression of TIM-3 (58)
	*rs4704853G>A* *(-822C>T)* *rs10515746C>A* *(-574T>G)*		promoter region	
	*rs1036199* *(+4259T>G)*	*G*	may affect the mucin domain of TIM-3 (59)	
	*rs4704846A>G rs3087616T>C*		located in 3’UTR and have been predicted to be located in putative binding sites for miRNA and postulated as variants potentially involved in post-transcriptional regulation of HAVCR2 (60)	
***LAG-3***	*rs2365094G>C* *rs3782735G>A*		Intron Variants	

### *CTLA-4* Polymorphisms and Cancer Risk

The associations between *CTLA-4* polymorphisms and cancer were studied extensively. Five out of the aforementioned polymorphisms mainly focused researchers’ attention: *CTLA-4c.-1722T>C*, *CTLA-4c.-1661A>G*, *CTLA-4c.-319C>T*, *CTLA-4c.49A>G*, and *CTLA-4CT60G>A*. The association of these polymorphisms with susceptibility to various types of cancers were investigated in many case-control studies and their results were analyzed in several meta-analyses. In the next paragraphs the reported relationships between genetic variants of the *CTLA-4* gene and particular cancers were described.

### Overall Cancer Analysis

Several meta-analyses combining data from different studies have been performed over the recent years. The most current meta-analysis by Fang et al. (61) included 67 different studies analyzing *CTLA-4c.49A>G* (23,617 cases, 27,261 controls), *CTLA-4c.-319C>T* (7,741 cases, 9,611), *CTLA-4CT60G>A* (9,675 cases, 9,623 controls), *CTLA-4c.-1661A>G* (3,635 cases, 4,104 controls), and *CTLA-4c.-1722T>C* (in 14 studies). According to this analysis, three SNPs were significantly associated with overall cancer risk: *CTLA-4c.-1661A>G* (*G/G vs. A+*, OR = 1.38; *G+ vs. A/A* OR = 1.48); *CTLA-4c.-319C>T* (*T+ vs. C/C* OR = 1.33); *CTLA-4c.49A>G* (*A/A vs. G+*, OR = 1.1; *A+ vs. G/G* OR = 1.16), while *CTLA-4CT60G>A* and *CTLA-4c.-1722T>C* were not associated with overall cancer risk. In analyses stratified by ethnicity, both *CTLA-4c.49A>G* and *CTLA-4c.-1661A>G* were significant susceptibility polymorphisms in Asians, but not in Caucasians, while *CTLA-4c.-319C>T* was associated with overall cancer susceptibility in Caucasians (*T+ vs. C/C*, OR = 1.63). When stratify by cancer type, the strong associations between the *CTLA-4c.49A>G* and bone, liver and pancreatic cancers (*A/A vs. G+*, OR = 2.04, OR = 1.41 and OR = 1.67, respectively) as well as breast and head and neck cancers (*A+ vs. G/G*, OR = 1.27 and OR = 1.33, respectively) were observed. Additionally, *CTLA-4c.-1661A>G* was associated with significantly increased susceptibility to breast and head and neck cancers (*G+ vs. A/A*, OR = 1.44 and OR = 1.99, respectively). Previous meta-analyses by Geng et al. (2014), Sun et al. (2008) and Zhang et al. (2011) (38, 62, 63) demonstrated similar results. In particular, Geng et al. (62) showed that the possession of CTLA-4c.49*A allele, while Sun et al. and Zhang et al. (38, 63) that the possession of *CTLA-4c.49 A/A* genotype increased susceptibility to cancer development. The association of CTLA-4c.49*A allele with susceptibility to breast and lung cancers was noticed by Zhang et al. and Geng et al. (62, 63). When stratified by ethnicity the association seemed to be particularly relevant to Asians (62). On the contarary, the presence of CTLA-4c.-319*T allele was associated with an increased overall cancer risk exclusively in Europeans (63).

Three meta-analyses evaluated the relationship between *CTLA-4CT60G>A* and the susceptibility to overall cancer risk (63–65). The obtained results did not confirm association of *CTLA-4CT60G>A* with overall cancer susceptibility, but showed the association between this SNP and particular cancer types. The presence of *CTLA-4CT60 A/A* genotype increased the risk of skin cancer (65), while presence of CTLA-4CT60*G allele the risk of breast and cervical cancers (64). The results of previous studies also indicated association between CTLA-4c.-1661*G allele and an increased cancer risk, this observation was particularly relevant to gastric (65) and breast cancers (62, 65) in Asian population (65, 66). The results of latest meta-analysis (61) confirmed previous reports about lack of associations between *CTLA-4c.-1722T>C* and overall cancer risk (62, 67).

### Cervical Cancer

The most recent meta-analysis (68) summarizing the results of 11 studies (37, 69–78) (3,899 cases, 4,608 controls) indicated significant association between CTLA-4c.-319*T allele and *T/T* genotype and cervical cancer risk (*T/T vs. C+*, 1.96; *T+ vs. C/C* OR = 1.47, respectively). In a stratified analysis by ethnicity, a significant association was observed for CTLA-4c.-319*T allele in Asian, but not in Caucasian women. This meta-analysis confirmed the results of the previous studies (79, 80). Of note, in study by Pawlak et al. (73) the significant association between CTLA-4c.-319*T allele and G1 grade of tumor was found.

It has been conclusively established that *CTLA-4c.49A>G* is associated with the risk of cervical cancer. Several meta-analyses (79–81), including (71–76, 82), proved that the *A/A* genotype of *CTLA-4c.49A>G* and the possession of CTLA-4c.49*A allele confer the increased susceptibility to this cancer type of about 20% (OR = 1.20). In the study by Xiong et al. (365 women - cases and controls) the carriage of CTLA-4CT60*G allele was associated with increased cervical cancer risk (OR = 1.92), and with more advanced stages of this disease (37), whereas the other studies did not demonstrate such association (73, 75, 80).

The protective role of the *CTLA-4CT60 A/A* genotype was confirmed by a meta-analysis by Zhao et al. (64), who examined data from two reports (the carriage of CTLA-4CT60*G allele was associated with higher risk) (37, 75). However, due to a limited number of studies, future investigations are needed to establish the role of this SNP in susceptibility to cervical cancer. Collectively, the findings of presented studies suggest that women possessing CTLA-4c.49* A allele and CTLA-4c.-319*T allele are more prone to develop cervical cancer.

### Breast Cancer

The association between *CTLA-4c.49A>G* and breast cancer (BC) was evaluated in two meta-analyses (83, 84) including the data from 5 studies (38, 85–89). The obtained results demonstrated that the presence of CTLA-4c.49*A allele increased the susceptibility to BC (OR = 1.26). The most recent study examining relationship between *CTLA-4c.49A>G* and BC outcome was performed by Babteen and colleagues among Egyptian women (90). The authors demonstrated that women being the carriers of CTLA-4c.49*G allele were 1.8 times less likely to develop BC than women with *A/A* genotype. Moreover, the authors indicated the association between this SNP and nodal infiltration, metastasis, advanced clinical stages, and risk for recurrence. However due to limited number of patients within compared groups the result should be treated with caution. Additionally, this group explored pooled effect of *CTLA-4c.49A>G* in BC based on 9 studies, and found that the carriage of CTLA-4c.49*G allele decreased 1.3 times risk of BC, whereas the CTLA-4c.49*A allele conferred an increased risk of BC developing, what was in agreement with previous analyses (90).

The *CTLA-4c.-319C>T* was analyzed only in one meta-analysis performed by Chen et al. (84), based on three studies (86, 91, 92). This analysis indicated that the presence of the *CTLA-4c.-319 C/T g*enotype conferred susceptibility to BC (*C/T vs. C/C*, OR = 1.65).

Three meta-analyses: the meta-analysis by Zhao et al. (64) [comprised four papers (86–88, 93)], the meta-analysis by Dai et al. (83) [contained an additional study (89)], as well as the meta-analysis by Chen et al. (84) (the same studies as in Zhao et al. (64); examined the relationship between *CTLA-4CT60G>A* and the risk of BC. These studies revealed that *A/A* genotype was associated with lower risk of BC developing, while CTLA-4CT60*G allele increased the risk. Such association was not found in the study by Goske and colleagues (258 cases, 258 controls) (33), however the authors noted relationship between *CTLA-4CT60G>*A and tumor growth. The *G/G* genotype was associated with restricted tumor growth, while *G/A* and *A/A* genotypes promote tumor growth (33). This group also investigated relationship between *CTLA-4c.-658C>T* and BC and demonstrated that *C/C* genotype significantly increased the risk of BC development in comparison to *C/T* genotype (33). Moreover, the meta-analyses by Dai et al. (83) and by Chen et al. (84) revealed association between the CTLA-4c.-1661*G allele and susceptibility to BC development, while *CTLA-4c.-1772T>C* was not associated with BC risk in these studies.

### Gastric Cancer

The most recent meta-analysis published in 2020 (94) which relates to digestive system malignancies (among them gastric cancers) summarizes the results from available studies concerning the following *CTLA-4* SNPs: *CTLA-4c.-1772T>C*, *CTLA-4c.-1661A>G*, *CTLA-4c.-319C>T*, *CTLA-4c.49A>G*, *CTLA-4CT60G>A* and gastric cancer (GC) risk (38, 95–100). According to this meta-analysis, *CTLA-4c.49A>G* was not associated with GC risk, despite the fact that such association was demonstrated in some of individual studies included in this analysis. In contrast, examination of *CTLA-4c.-319C>T* revealed significant association between the possession of *CTLA-4c.-319 C/C* genotype and increased risk of GC (OR = 1.58). This finding is inconsistent with results for other types of cancer, where the CTLA-4c.-319*T allele has been identified as a tumor risk factor. Additionally, this meta-analysis indicated that the carriage of the CTLA-4c.-1661*A allele may predispose to GC development (OR = 1.78) (94).

### Hepatocellular Cancer

There is only a limited number of studies investigating *CTLA-4* gene polymorphisms and risk of hepatocellular cancer (HCC) available in the literature. One of such study was published in 2018 by Wang et al. (101), and analyzed data from four studies (82, 102–104). This meta-analysis showed that in the Chinese population the *CTLA-4c.49 A/A* genotype increased the risk of HCC of about 1.5-fold. On the other hand, the most recent meta-analysis by Li et al. (94) [analyzing data included in work by Wang et al. (101), except one study published in Chinese language (104), and data from two additional studies (105, 106)], did not confirm previous observations and suggested a lack of association between the *CTLA-4c.49A>G* and HCC risk.

Yang et al. (105) investigated association between *CTLA-4c.49A>G*, *CTLA-4CT60G>A*, *CTLA-4c.-1722T>C*, *CTLA-4 rs16840252C>T*, and HCC risk in a group of 584 patients and 923 control subjects of an Eastern Chinese Han population. This study revealed that the carriers of CTLA-4CT60*A allele were about 1.5 times more prone to develop HCC than individuals being homozygotes *G/G* (105). An opposite association was obtained by Wang et al. (106) on 554 HCC patients and 612 control subjects from Chinese population. The authors observed about 1.5 increased risk of HCC cancer for individuals with *CTLA-4CT60 G/G* genotype as compared to carriers of CTLA-4CT60*A allele. Additionally, this study demonstrated that carriers of CTLA-4-319*T allele had 1.6 times higher risk of HCC in comparison to individuals with *C/C* genotype, this risk increased to 2.6 in case of *T/T* homozygotes. Similarly, to results obtained by Xiong and colleagues (37), these authors observed that the *CTLA-4c.-319 C/T* and *T/T* genotypes were associated with lower production of interleukins IL-2 and IL-4, an increased production of TGF-β and lower cell proliferation, what was demonstrated for PBMCs with these genotypes stimulated with PHA. These cells were also less cytotoxic to HepG2 liver cancer cells (106).

### Colorectal Cancer

The evaluation of association between *CTLA-4* polymorphisms and colorectal cancer (CRC) risk was the subject of several studies.

The meta-analysis by Jiang et al. (2013) (107) [based on data from (96, 108–110)] did not show association between the *CTLA-4c.49A>G* and CRC risk. Importantly, most of the studies included in this work were performed on Caucasians.

Two meta-analyses: one by Wang et al. (2015) (111) (7 studies (96, 108, 110, 112) and 2 articles in Chinese) and another performed by Zhang et al. (2018) (113). (9 common studies (96, 108, 110–112, 114) and 4 articles in Chinese) as well as 2 additional studies (109, 115) showed that the possession of the CTLA-4c.49*G increased the risk of CRC about two-fold (111). Moreover, Zhang et al. (113) established that *A/A* genotype protected against CRC in the Asian population.

The most recent meta-analysis by Li et al. (94) [analyzing data from 8 out of 11 reports included in paper by Zhang et al. (113)] reported that *CTLA-4c.49 G/G* genotype decreased risk of colorectal cancer about 1.8 times in comparison to *G/A* and *A/A* genotypes. Due to discrepancies observed in aforementioned publications future well designed studies are needed to resolve ambiguities between published results. For *CTLA-4c.-1722T>C*, *CTLA-4c.-1661A>G*, *CTLA-4c.-319C>T*, and *CTLA-4CT60G>A*, there was no evidence of associations with CRC risk (94, 114).

### Bone Cancer

In 2015 Fan at al. (116) published results of meta-analysis exploring association between *CTLA-4c.-1661A>G*, *CTLA-4c.49A>G*, *CTLA-4CT60G>A*, and the risk of bone sarcoma based on data from four case-control studies (117–120) performed on Asians. The authors demonstrated that *CTLA-4c.49 A/A* genotype increased 2 times risk of bone sarcoma development. The other SNPs were not associated with the risk of this cancer.

The relationship between *CTLA-4c.-319C>T* and *CTLA-4c.49A>G* and susceptibility to osteosarcoma was examined most recently in a meta-analysis by Wang et al. (121) (based on 3 reports: Wang et al. (117, 118), Liu et al. (117) and Qiqo et al. (study published in 2016 in Chinese a detailed reference was not provided in publication). The authors showed that CTLA-4c.49*A allele as well as CTLA-4c.-319*T allele conferred about 2-fold increased risk of developing osteosarcoma in Chinese population. Bilbaoilbao-Aldaiturriaga and colleagues (122) investigated association between *CTLA-4c.49A>G* and osteosarcoma (99 patients, 125 controls) and concluded that carriers of CTLA-4c.49*G allele had 2-fold lower risk of osteosarcoma in comparison to homozygotes *A/A* in Spanish population. They also performed a meta-analysis based on their results and 2 other studies performed on Chinese population (117, 118), and concluded that in spite of different frequency of CTLA-4c.49*A allele between Spanish and Chinese population (63 *vs*. 33 and 35%) the *A* allele and *A/A* genotype were associated with 1.36 and 2.07 times higher risk of osteosarcoma in both populations (122). Collectively, the results of these studies suggest that *CTLA-4c.49 A/A* genotype increased about 2 times risk of osteosarcoma development.

### Hematological Malignances

The associations of *CTLA-4* polymorphisms and susceptibility to hematological malignances were relatively frequently investigated in Asian and Caucasian populations.

Dai and colleagues (123) performed a meta-analysis [based on data from 9 case-control studies performed on Caucasians (7 reports) and Asians (2 reports) (95, 124–131)] aimed at evaluation of association between *CTLA-4c.-319C>T*, *CTLA-4c.49A>G*, *CTLA-4CT60G>A*, and susceptibility to lymphoid malignancies. None of investigated SNPs were associated with overall risk of developing lymphoid malignancy. Furthermore, a stratified analysis by ethnicity (Asian or Caucasian) and histopathological subtype (non-Hodgkin lymphoma) also failed to detect an association between the studied polymorphisms and risk of lymphoid malignancy. Hui et al. (132) examined *CTLA-4c.-319C>T*, *CTLA-4c.49A>G*, and *CTLA-4CT60G>A* SNPs in childhood acute lymphoblastic leukemia (ALL) and demonstrated that the presence of CTLA-4c.-319*T allele conferred susceptibility to this disease (132). The other SNPs were not associated with risk of ALL in children.

### Lung Cancer

There are a very limited number of studies on the association between the *CTLA-4* polymorphisms and lung cancer risk. Furthermore, available studies demonstrated inconsistent results, and they were not collectively analyzed recently. The only published so far meta-analysis (62) [including data from three studies (38, 133, 134)] showed that the possession of CTLA-4c.49*A allele slightly increased risk of lung cancer (1.2-fold). Similar results were obtained in meta-analyses by Zhang et al. (63) and Geng et al. (62), however in the study by Geng et al. (62) this association was restricted only to Asians. Two other investigated SNPs of *CTLA-4*, namely *CTLA-4c.-1722T>C* and *CTLA-4c.-1661A>G* were not associated with lung cancer risk (62). The association between carriage of CTLA-4c.49*A allele and increased risk of lung cancer was not confirmed in two other studies (135, 136). The *CTLA-4c.-319C>T* (133, 136) and *CTLA-4CT60G>A* (133) were not associated with the risk of lung cancer development.

### Urological Cancers

The literature data on urological cancers are limited. There is only one study considering associations between *CTLA-4c.49A>G* and *CTLA-4CT60G>A* and bladder cancer risk in North Indian population (200 cases, 200 controls) (137). The authors found that the *G/G* genotype of *CTLA-4c.49A>G* was associated with almost 4-fold higher risk of bladder cancer in comparison to *A/G* and *A/A* genotypes. This group also demonstrated that *CTLA-4CT60 G/G* genotype increased almost 1.4 times susceptibility to develop bladder cancer (137).

The relationship between *CTLA-4c.-319C>T*, *CTLA-4CT60G>A* and prostate cancer (PC) (301 cases, 301 controls) was investigated by Karabon et al. The authors observed overrepresentation of the carriers of the CTLA-4c.49*A allele and the carriers of the CTLA-4c.-319*T allele in patients as compared to controls. Moreover, the individuals possessing both susceptibility alleles CTLA-4c.49*A and CTLA-4c.-319*T had 1.78-fold increased risk of PC than individuals with protective *G/G* and *C/C* genotypes, respectively (138).

Two studies analyze relationship between *CTLA-4* SNPs and renal cell cancer (RCC) risk and reported results contradicting each other (109, 139). In the Spanish study (127 cases, 176 controls) (109) the *CTLA-4c.49 A/A* and *CTLA-4CT60 A/A* genotypes were associated with about 2-fold increased risk of RCC. Additionally, the authors observed overrepresentation of this genotypes in RCC patients, particularly with higher grade tumors. In Polish case-control study (323 patients, 518 controls) the following SNPs: *CTLA-4c.49A>G*, *CTLA-4c.-319C>T*, *CTLA-4CT60G>A* were investigated. The authors reported that the presence CTLA-4CT60*G allele increased 1.5 times risk of clear cell renal cancer. Furthermore, the presence of CTLA-4CT60*G allele was significantly associated with necrosis and advanced stages of a clear cell renal cell cancer (139). Due to inconsistent results further study are needed to resolve the ambiguity.

### Pancreatic Cancer

Only two studies evaluated the potential association between *CTLA-4* polymorphisms and pancreatic cancer (PC) risk. Lang et al. (140) examined association between *CTLA-4c.49A>G* and PC and reported that CTLA-4c.49*A allele conferred the risk of PC and that the homozygotes *A/A* were 2.2 times more prone to develop PC in comparison to homozygotes *G/G* and heterozygotes *A/G*. The second study by Yang et al. (141) confirmed this observation by demonstrating that the homozygotes *A/A* had 2.5-fold increased risk of PC development (141). The most recent meta-analysis, mentioned in part overall cancer risk, by Fang et al. (2018) confirmed that homozygotes *A/A* were more prone to develop PC (OR = 1.67). Based on these findings the CTLA-4c.49*A allele may be considered as risk factor of PC. The summary of above presented associations between *CTLA-4* polymorphisms and cancer risk is shown in **Supplementary Table 1**.

## Programmed Cell Death Protein 1

PD-1 was identified in 1991 at Kyoto University by a group led by Tasuku Honjo, and the first article about this molecule was published one year later (2). In 1999, the same research team demonstrated the role of the PD-1 molecule—negative regulation of the immune response (142). Finally, three years later was shown that the blockade of interaction between PD-1 and its ligand (PD-L1) may provide a promising strategy for cancer immunotherapy (143).

**Figure 2:**
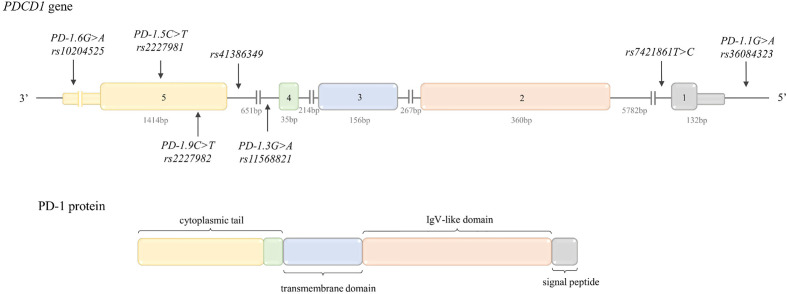
Structure of *PDCD1* gene and protein. Top: *PDCD1* gene structure. The figure shows the polymorphisms described in the review and the lengths of the exons and introns. Bottom: PD-1 protein structure. The colors indicate which region of the protein is encoded by which exon.

PD-1 (CD279) is a type I transmembrane protein belonging to the immunoglobulin (Ig) superfamily (**Figure 2**). The expression of PD-1 is strictly and dynamically regulated. Low basal levels of PD-1 are observed on resting naïve T cells as well as in certain populations of developing thymocytes (which has been linked to immune tolerance). After immune stimulus, PD-1 can be transiently expressed on CD4^+^ and CD8^+^ T cells, B cells, macrophages and its expression can be also detectable on natural killer T (NKT) cells and some subset of dendritic cells (DCs) (44, 144, 145). Downregulation of PD-1 is noticed during acute antigen exposure, whereas in the case of chronic immune stimulation (e.g. when T cells are exposed persistently to tumor cells) PD-1 is overexpressed (144). This high level of PD-1 expression can lead to functional impairment - tumor resident T cells frequently present signs of exhaustion.

PD-1 binds two different ligands—PD-L1 (CD274, B7-H1) and PD-L2 (CD273, B7-DC). PD-L2 affinity for PD-1 is 3-fold higher than PD-L1; however, PD-L1 is expressed on more cell types than PD-L2 (44). Like in the case of CTLA-4/CD80, PD-1/PD-L1 interactions play an opposite role to CD28-CD80 costimulatory pathway (146).

In normal tissue, binding of PD-L1 by PD-1 has been shown to play a pivotal role in maintaining immune homeostasis and prevention from autoimmunity during infection and inflammation (147). In the tumor microenvironment (TM), interaction between PD1 and PD-L1 (expressed on tumor cells) provides an immune escape mechanism for tumor cells (147).

Given the tumor immunology, PD-1 mainly inhibits T cell activation in TM at later stages of tumor growth, whereas signaling *via* CTLA-4 regulates T cell activation in the early stage of T cell response in the lymph nodes (LNs) (147).

PD-1 is encoded by the *PDCD1* gene (also known as *PD-1*) located on chromosome 2q37.3 in reverse (REV) orientation (148). The gene structure is presented in **Figure 2**.

Regulatory elements of *PDCD1* expression are located *inter alia* in two conserved regions 100bp and 1.1kb upstream of the transcription start site (TSS). They contain multiple TFs binding sites: activator protein-1 (AP-1) binding site, interferon-stimulated response element (ISRE), as well as binding sites for nuclear factor of activated T cells (NFATc1), a FoxO1, NF-κB and Notch. Two additional regulatory elements (-3.7 and +17.1kb form the TSS) contain STAT binding sites and contribute to the enhanced transcriptional activity after TCR and IL-6 or IL-12 cytokine stimulation. The mammalian transcriptional insulator CCCTC-binding factor (CTCF) is bound by elements located at -26.7 and +17.5kb that form constitutively interacting chromatin loops (144).

### *PDCD1* Gene Polymorphisms

**Table 1** includes the frequency of *PDCD1* polymorphisms described below in different populations.

***rs36084323C>T* (*PD-1.1, G>A* REV)** is located in the promoter, in the putative binding site for the UCE-2 transcription regulators. According to literature (149), promoter activity in the construct containing PD-1.1*G allele was significantly higher than that with the PD-1.1*A allele. Given the above, one can assume that carriers of PD-1.1*G allele could have a higher expression of PD-1 and in consequence inhibited activation and proliferation of T cells, which in turn can lead to poor ability to fight/remove cancer cells (41). However, some literature data [among others: (41, 150, 151)] described the PD-1.1*G allele as associated with a decreased risk of cancers (detailed description below) and therefore further functional studies are necessary.

***rs11568821C>T* (*PD-1.3, G>A* REV)** is located in intron 4, in an enhancer-like structure where four imperfect tandem repeats are placed. This region contains binding sites for TFs involved in hematopoietic differentiation and inflammation (among others RUNX1, E-box–binding factors and NFκB1). According to Prokunina et al. (43) the presence of PD-1.3*A allele disrupted the binding site for RUNX1 TF in the first repeat, which in consequence may lead to aberrant PD-1 expression and deregulated lymphocyte activity (44).

***rs2227981G>A* (*PD-1.5*, *C>T* REV)** is located in exon 5. This synonymous variation (Ala268Ala) does not modify the amino acid structure of the protein. Therefore, it is speculated that this SNP may be in LD with another polymorphism, which may alter PD-1 expression (44). It was shown that PD-1 expression (% PD-1^+^CD4^+^ T cells) was significantly lower in individuals with *PD-1.5 C/C* genotype than those with the *PD-1.5 C/T* and *PD-1.5 T/T* genotypes (46). From the aforementioned observation it may be concluded that subjects with *PD-1.5 C/C* genotype could have lower risk of cancer development.

***rs10204525C>T* (*PD-1.6*, *G>A* REV)** is located in the 3’UTR region, in a putative miRNA binding site. According to a study by Zhang et al. (47) miR-4717 may allele-specifically regulate PD-1 expression. In lymphocytes from chronic HBV patients with *PD-1.6 G/G* genotype (but not in lymphocytes from patients with *PD-1.6 A/A*) miR-4717 mimic significantly decreased PD-1 expression and increased TNF-α and IFN-γ production, whereas miR-4717 inhibitor acted in opposite way (47). Moreover, Zhang et al. showed that *PD-1* mRNA levels were the highest in individuals with *PD-1.6 A/A* genotype and decreased as the number of *G* allele increased; in HBV patients PD-1 expression in individuals with *PD-1.6 G/G* genotype was significantly lower in comparison to subjects with *PD-1.6 A/A* genotype (48). Since inhibition of PD-1 promotes an effective immune response against cancer cells (152), PD-1.6*G could be considered as protective allele in cancer development.

***rs2227982G>A* (*PD-1.9*, *C>T* REV)** is a nonsynonymous SNP, located in exon 5, causing an amino acid substitution from alanine to valine in the extracellular domain of PD-1 leading to a different structure and possibly altering the function of PD-1 (42, 44).

***rs7421861A>G* (*T>C* REV)** is located in intron 1, where a number of regulatory elements and splicing control elements exist. Due to the disruption of the splice site or alteration of the mRNA secondary structure, PD-1 *rs7421861T>C* may induce aberrant splicing, and lead to translational suppression (42).

***rs41386349G>A* (*C>T* REV)** is located in intron 4. According to data presented by Zheng et al. (45) this SNP is placed in a putative enhancer-like region. Rs41386349*T allele created a negative cis-element for transcription and had lower *PD-1* transcriptional activity in human T cells than rs41386349*C allele (45). Therefore, it can be concluded that rs41386349*T allele conferring higher risk for autoimmune diseases could be considered as protective factor against cancer development. The possible functional relevance of *PDCD1* SNPs was summarized in **Table 2**.

### *PDCD1* Polymorphisms and Cancer Risk

#### Overall Cancer Risk

Recent comprehensive meta-analysis regarding the association of *PDCD1* polymorphisms with overall cancer risk was performed by Hashemi et al. in 2019 (153). Having regard to 16 studies (5,622 cases, 5,450 controls) (71, 136, 150, 151, 154–163) the authors concluded that the *PD-1.5* was associated with susceptibility to cancer development, with the *PD-1.5 T/T* genotype decreasing the overall risk (OR = 0.82). A similar conclusion concerning *PD-1.5* was drawn by the authors of three earlier meta-analyses (42, 164, 165). Admittedly, in the literature there is one meta-analysis (166) that did not show association between *PD-1.5* and total cancer risk, however, according to our best knowledge that was the first meta-analysis regarding *PD-1.5*, performed on relatively small number of subjects (1,427 patients, 1,811 controls) (166).

The findings of the meta-analysis by Hashemi et al. (153) revealed that in addition to *PD-1.5*, also *PD-1.3* was associated with overall cancer risk. On the basis of 9 articles (136, 154, 156, 157, 160, 167–170) (1,846 cases, 1,907 controls), a decreased risk of cancer was observed for carriers of PD-1.3*A allele (OR = 0.82). Two earlier meta-analyses: by Zhang et al. (164) and by Dong et al. (42) (in the case of the *PD-1.3*, both including the same 4 studies) indicated an association of *PD-1.3 G/A* genotype with lower overall cancer risk.

Furthermore, the results of the latest meta-analysis (3,576 patients, 5,277 controls) (153) revealed that the carriers of *T/C* genotype of *rs7421861T>C* had an increased risk of cancer (OR = 1.16). Given the results of this study, it can be assumed that higher risk of cancer concerned carriers of rs7421861*C allele (OR = 1.14) (153). Despite the results of two previous meta-analyses (42, 164) being similar to that obtained by Hashemi et al. (153) significant differences for *rs7421861T>C* were not observed, probably, due to the small effect size of this polymorphism and a small number of patients and controls included in both meta-analyses.

No evidence of association between *PD-1.1* and overall cancer risk was observed in two additional meta-analyses (41, 153). However, analysis stratified by ethnicity (performed as part of the meta-analysis by Da et al., 4,445 cases, 5,126 controls), revealed an increased risk of cancer for individuals with *PD-1.1 A/A* genotype in the Asian population (OR = 1.15). Of note, taking into consideration the completely different genotype distribution among populations (**Table 1**), in the case of this polymorphism it would be more appropriate to perform analyses stratified by ethnicity. No associations with overall cancer risk were found for the remaining SNPs of the *PDCD1* gene (quite broadly examined in the context of cancers)—*PD-1.9* (42, 153, 164) and *PD-1.6* (153, 164).

#### Cervical Cancer

As far as we know, only *PD-1.5* SNP was investigated in the context of cervical cancer. Ivansson et al. (71), based on the study conducted on 1,306 patients and 811 controls, reported the *PD-1.5 T/T* genotype as being associated with reduced cervical cancer susceptibility (OR = 0.69). Whereas, in the study by Li et al. (256 cases, 250 controls) (158) *PD-1.5 C/T* genotype was indicated as being associated with an increased risk of cervical cancer (OR = 2.18). However, given the fact that in the study by Li et al. genotype distributions both in controls and cases were not in HWE, these results should be treated cautiously. In the study by Guzman et al. (171) *PD-1.5* separately was not associated with susceptibility to cervical cancer. The association with increased risk was observed only for a combination of genotypes *CD28*(rs3116496TT)/*IFNG* (rs2430561AA)/*PDCD1*(rs2227981CT), although a major contribution to the observed association was suggested for *CD28* and *IFNG* SNPs.

#### Breast Cancer

Polymorphisms of the *PDCD1* have been studied also in breast cancer. Two meta-analyses (153, 165) investigating the association between *PDCD1* SNPs and overall cancer risk, in a subgroup analysis by cancer type (951 patients, 806 controls), indicated an association between *PD-1.5* and susceptibility to BC. A decreased risk of BC (OR = 0.8) was observed for carriers of the PD-1.5*T allele (increased for *PD1.5 C/C* genotype). This observation is in line with the results obtained for overall cancer risk, however it is worth mentioning that two subgroup analyses for BC (performed as part of meta-analyses) were conducted on the same two studies. Two original research articles on the basis of which the analyses were made (150, 157), presented inconsistent results. Namely, from the results of the study by Hua et al. (486 patients, 478 controls) (150) one can conclude that carriers of the PD-1.5*T allele had lower susceptibility to BC, OR = 0.68 (however the distribution of genotypes in the control group was not in Hardy-Weinberg equilibrium although the manuscript stated otherwise), whereas in the study by Haghshenas et al. (435 patients, 328 controls) (157) there was no evidence indicating association between the *PD-1.5* and susceptibility to BC. Conflicting results were also noted for the *PD-1.9*. Hua et al. (150) suggested no associations of this SNP with BC risk, just as in the case of overall cancer risk. However, Ren and colleagues (172) in the study conducted on 560 patients and 583 individuals observed a decreased risk for BC in individuals carrying the PD-1.9*T allele (OR = 0.69). A similar association was observed in a subgroup analyses by cancer type (combining the results of two above mentioned studies) performed by Hashemi et al., as part of meta-analysis (153). Apart from *PD-1.5* and *PD-1.9*, also *PD-1.1* was investigated in the context of BC. This SNP was considered to be associated with susceptibility to BC. Increased risk of BC was identified for PD-1.1*A allele carriers (decreased for *PD-1.1 G/G* genotype, OR = 0.71) (150), which is in accordance with the results obtained for overall cancer risk in Asians. No association with breast cancer risk was suggested for *rs7421861T>C* (150, 153, 172), *PD-1.6* (172), and *PD-1.3* (157).

#### Ovarian Cancer

According to our best knowledge, only two studies (151, 173) have assessed the potential association between *PDCD1* polymorphisms and ovarian cancer. The results of a case-control study (164 patients, 170 controls) by Tan et al. demonstrated that possession of PD-1.9*T allele was associated with increased risk of ovarian cancer (OR = 1.67). The same allele was also associated with higher FIGO stage and higher differentiation grade (173). It is worth recalling here that in the case of BC, it was showed (172) that the PD-1.9*T allele was associated with decreased risk. Furthermore, Li et al. (151) reported that the *PD-1.1* is associated with susceptibility to ovarian cancer, with increased risk for individuals with *PD-1.1 A/A* genotype (decreased risk for subject with PD-1.1*G allele carriers, OR = 0.70), as in the case of overall cancer risk in Asians. Additionally, from data obtained by Li et al. it can be concluded that the PD-1.5*T allele was associated with a reduced risk of ovarian cancer (OR = 0.82) (151).

#### Gastrointestinal Cancers

Stratified analysis carried out as part of the meta-analysis by Hashemi et al. mentioned above (153) was aimed *inter alia* at evaluation of association between *PDCD1* polymorphisms and the risk of gastrointestinal cancer (a cancer group that affects the digestive system which contains *inter alia* esophageal cancer, esophagogastric junction adenocarcinoma, gastric cancer, hepatocellular carcinoma and colorectal cancer, which are separately described below). Just as in the case of overall cancer risk, decreased risk of gastrointestinal cancer was observed for individuals with the *PD-1.5 T/T* genotype (OR = 0.60), whereas increased risk was noted for subjects with *rs7421861 T/C* genotype (OR = 1.19) (153). A higher susceptibility to gastrointestinal cancer was also identified for carriers of PD-1.9*T allele (OR = 1.16) (153). It is noteworthy that similar association was observed for ovarian cancer, however no evidence of association was found between *PD-1.9* and overall cancer risk.

As for particular types of gastrointestinal cancer, according to our best knowledge, in the literature there are only three studies (163, 174, 175) aimed at investigating the *PDCD1* SNPs as potential risk factors for esophageal cancer, more precisely esophageal squamous cell carcinoma (ESCC). In all three studies the *PD-1.6* was suggested to be associated with susceptibility to esophageal cancer. However, two of them (163, 174) showed a decreased risk for *PD-1.6 G/G* individuals (OR = 0.59 and OR = 0.68, respectively) (in the study by Qiu et al. (174) deviation from HWE was observed in controls), while Zang et al. (175) reported the opposite effect—carriage of PD-1.6*G allele was associated with increased risk of esophageal cancer (OR = 1.26). Moreover, in this study individuals with *PD-1.6 G/G* genotype and carriers of PD-1.6*G allele had, respectively, higher TNM stage (OR = 1.81) and higher risk of distant metastasis (OR = 1.67) (175). Taking into consideration the inconsistent results presented above as well as the fact that there was no evidence of association between this SNP and overall cancer risk—more research on this issue is needed. The results regarding the *rs7421861T>C* are also inconsistent. Some evidence suggests increased risk of esophageal cancer (OR = 1.24) and higher TNM stage (OR = 1.37) for rs7421861*T allele carriers (decreased for individuals with *rs7421861 C/C* genotype) (175) while another shows no association of this SNP with ESCC at al. (174). As was described above, analysis conducted for gastrointestinal cancers pointed to association between rs7421861*C allele with increased risk. Due to such inconsistent results, further study will be required in order to solve this issue. For the *PD-1.9* no association with ESCC susceptibility was found in two studies for overall population (163, 174), however, the *PD-1.9 C/T* genotype was pointed out as associated with increased ESCC risk in females (OR = 1.71) (163). Moreover, the *PD-1.5 C/T* allele was associated with increased ESCC risk in the group of smokers (OR = 1.48) (163). This observation is not in line with the results of analysis regarding gastrointestinal cancers and overall cancer risk, demonstrating association of the *PD-1.5 T/T* genotype with decreased susceptibility. Also, the *PD-1.1* was investigated in the context of ESCC (163, 175), however, there was no evidence for association between this SNP and susceptibility to ESCC.

To the best of our knowledge, only Tang et al. (176) made an attempt to find a potential association between *PDCD1* polymorphisms (*PD-1.1*, *PD-1.6*, *PD-1.9*, and *rs7421861T>C*) and risk of esophagogastric junction adenocarcinoma (EGJA) (however it is worth mentioning that 1,063 patients and 1,677 controls were enrolled in this study). Increased risk of EGJA (similarly as in the case of overall cancer risk) was noted for carriers of rs7421861*C allele (OR = 1.43) (176). Higher susceptibility to EGJA was also observed for carriers of the PD-1.9*T allele (lower for the *PD-1.9 C/C* genotype, OR = 0.81) and this observation was in accordance with the results obtained for gastrointestinal cancer (153), ESCC in females (163) as well as for ovarian cancer (173). Moreover, according to the authors’ suggestion, for individuals with the *PD-1.1 C/C* decreased risk of EGJA was noted (OR = 0.86) (176). No association with susceptibility to EGJA was found for the *PD-1.6* (176).

Savabkar et al. in their study aimed at determining the potential association between *PD-1.5* and gastric cancer indicated that the presence of *PD-1.5 C/T* genotype may be a risk factor for GC (OR = 1.77) (161). From this study one can conclude, that increased risk of GC concerned PD-1.5*T allele carriers. However, these observations are in opposition to that for overall cancer, for gastrointestinal cancers as well as for other types of cancer (breast, cervical, ovarian). Given the inconsistency with the results of other research and the fact that only 122 GC patients and 166 controls were enrolled to this study, further examination of this SNP in the context of GC will be required.

The *PD-1.9*, *PD-1.6*, and *rs7421861T>C* were studied in gastric cardia adenocarcinoma (GCA) (177), however, for none of them the difference in genotype distribution between 330 GCA patients and 608 controls were found. The authors indicated a possible association between the presence of PD-1.9*T allele with increased risk of GCA among ever drinking cases (OR = 2.04) (177).

According to our best knowledge, only Bayram et al. (167) and Li et al. (178) made an attempt to find out whether *PD-1.3* and *PD-1.6* may be considered as risk factors for HCC. There was no evidence of association between the *PD-1.3* and HCC risk in the study carried out by Bayram et al. on 236 patients and the same number of control subjects (167). However, for individuals with *PD-1.6 A/A* genotype, Li et al. in the study conducted on 271 patients and 574 non-HCC subjects observed a higher susceptibility to HCC (OR = 1.47) (178).

Genetic variations in the *PDCD1* were also investigated in colorectal cancer (54, 159, 170, 179). No evidence of association between *PD-1.5* and overall CRC risk was found in the study (200 patients, 200 controls) by Mojtahedi et al. However, after patients subdivision for cancer location, it was observed that *PD-1.5 C/T* genotype was significantly more frequent in colon cancer patients as compared with healthy controls (OR = 1.74) (159). Moreover, decreased risk of colon cancer was observed for PD-1.1*A allele carriers (OR = 0.09) (179). However, taking into consideration the sample size (76 patients, 73 controls), this result should be treated with caution (especially having in mind the inconsistency in the results for other cancers). Of the four SNPs (*rs6710479, rs7421861T>C, PD-1.9, PD-1.6*) examined by Ge et al. (601 patients, 627 controls), only *rs7421861T>C* was shown to be associated with susceptibility to CRC, with the *rs7421861 T/C* genotype increasing risk of CRC development (OR = 1.31) (54). Additionally, from the study conducted by Yousefi et al. it can be concluded that the PD-1.3*A allele was associated with increased CRC risk (OR = 2.18) (170), however, it should be stated that only a small number of patients and controls (80 and 110, respectively) were enrolled in this study and further investigations will be needed. Furthermore, it is also noteworthy to mention that in the group of Korean patients (N = 688), individuals with *PD-1.6 A/A* genotype had shorter overall survival as compared to PD-1.6*G allele carriers (HR = 1.47) (180).

#### Hematological Malignancies

The potential association of *PDCD1* polymorphisms with hematological malignancies was evaluated in several studies including multiple myeloma and leukemia.

According to Kasamatsu et al. (181), none of the three investigated SNPs (*PD-1.1, rs41386349, PD-1.9*) individually were associated with multiple myeloma (MM). However, the authors observed higher frequency of the haplotype combination (*PD-1.1 rs41386349 PD-1.9/PD-1.1 rs41386349 PD-1.9*) G C C/G C C in the group of MM patients (N = 124) as compared with controls (N = 211). In the study by Grzywnowicz et al. (182), no associations between five SNPs of *PDCD1* (*PD-1.1, PD-1.3, PD-1.5, PD-1.9*, and *rs41386349C>T*) and susceptibility to chronic lymphocytic leukemia (CLL) were found in the group of 114 patients and 150 controls (182). Ramzi et al. reported the *PD-1.9 C/T* genotype as associated with a decreased risk of leukemia (OR = 0.43) (169), however, taking into consideration the fact that only 59 leukemia patients (28 acute myeloid leukemia, 20 acute lymphocytic leukemia, 11 chronic myelogenous leukemia) and 46 controls were enrolled in this study, these results should be interpreted warily.

#### Non-Small Cell Lung Cancer

The evaluation of potential associations between the *PDCD1* SNPs and lung cancer was performed only in few studies. In a subgroup analysis performed on 1,058 patients and 1,103 controls [being part of the above mentioned meta-analysis by Hashemi et al. (153)] carriage of PD-1.5*T allele was associated with decreased risk of lung cancer (OR = 0.84). It is worth noting, that this meta-analysis was performed on the basis of three studies on lung cancer (136, 154, 162), two of which did not show association (136, 154). In two of them (136, 162) the distribution of genotypes in the control group was not in Hardy-Weinberg equilibrium (although the manuscripts state otherwise). Of note, the *PD-1.5 T/T* genotype decreased the risk of advanced NSCLC (TNM stage III and IV). Lack of association with non-small cell lung cancer (NSCLC) was found for the remaining studied polymorphisms *PD-1.1* (136, 183), *PD-1.3* (136, 154), and *PD-1.9* (136).

#### Other Cancers

According to our best knowledge, there are only individual articles regarding *PDCD1* polymorphisms in relation to thyroid cancer (156), head and neck squamous cell carcinomas (168), brain tumor (160), cutaneous melanoma (155) and basal cell carcinoma (184). It was found that PD-1.5*T allele was associated with increased risk of thyroid and brain cancers, while PD-1.3*A allele with decrease risk of basal cell carcinoma. However, most of the studies were conducted on small groups of patients and controls, therefore further studies will be required to verify and confirm described associations. **Supplementary Table 2** shows the summary of above presented associations between *PDCD1* SNPs and cancer risk.

## Programmed Death Ligand 1

Programmed death ligand 1 (PD-L1) was discovered in 1999 by Chen’s group, but at first it was not identified as PD-1 ligand (185). One year later, a group led by Gordon Freeman collaborating with the Genetics Institute at Cambridge established that the newly discovered molecule was a PD-1 ligand. Engagement of PD-1 with this newly identified molecule decreased the proliferation and cytokine production of T cells upon stimulation with anti-CD3 antibody. Then it became clear that PD-1/PD-L1 pathway prevents autoimmunity by inhibiting the activation of T cells (186, 187).

**Figure 3:**
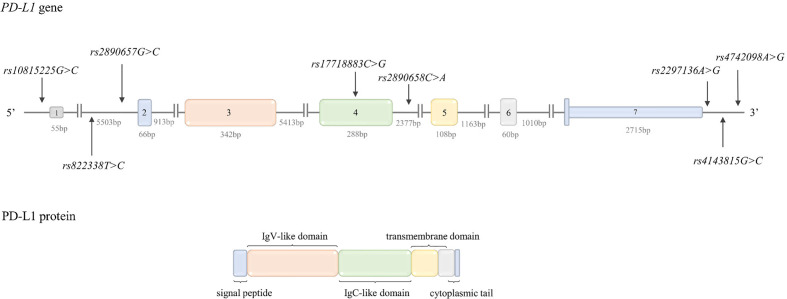
Structure of PD-L1 gene and protein. Top: *PD-L1* gene structure. The figure shows the polymorphisms described in the review and the lengths of the exons and introns. Bottom: PD-L1 protein structure. The colors indicate which region of the protein is encoded by which exon.

PD-L1, similarly to PD-1 is type I transmembrane protein (consisting of 290 aa) and belongs to the Ig superfamily (**Figure 3**). PD-L1 expression can be constitutive or inducible. Under physiological conditions, PD-L1 is expressed constitutively at a low level on T cells, B cells, DCs, monocytes, mesenchymal stems cells, bone marrow derived mast cells, vascular endothelial cells, keratinocytes, pancreatic islet cells, astrocytes as well as various immune privileged tissues and organs (such as the placenta, testis and the anterior chamber of the eye), where exogenous antigens are tolerated without induction of inflammation and/or infection (145, 188, 189). In the context of inflammation and/or infection PD-L1 expression can be induced on hematopoietic, endothelial, and epithelial cells. PD-L1 can be also expressed by tumor cells and tumor stroma (188). Therefore, the interaction between PD-1 and PD-L1 results in an activation of self-tolerance pathways not only in immune cells but also in tumor cells, and in this way provides an immune escape mechanism for the tumor (145).

There are two mechanisms by which tumor cells can express PD-L1: 1) “innate immune resistance” which refers to constitutive PD-L1 expression on tumor cells. This type of expression can be the result of *inter alia PD-L1* gene amplification or aberrant activation of oncogenic signaling pathways and 2) “adaptive immune resistance” which refers to PD-L1 expression in response to inflammatory factors secreted in the TM during the immune response against a tumor (189).

PD-L1 is encoded by the *PD-L1* gene (*PDCDL1, CD274*) located on chromosome 9p24.1. The gene structure is shown in **Figure 3**. *PD-L1* transcriptional activation is regulated by such transcription factors as: STAT3, MYC, NF-κB, AP-1, and HIF-1 (147, 188). The mechanisms regulating PD-L1 expression are described in detail in a review by Sun et al. (188).

### *PD-L1* Polymorphisms

The frequency of the *PD-L1* polymorphisms (described below) in different populations is presented in **Table 1**.

***rs4143815G>C*** is located in 3’UTR. *In silico* analysis predicted that *rs4143815G>C* is situated in putative binding site for miR-7-1^*^, miR-495, miR-298 (51) and miR-570 (51, 52). According to Wang et al. (52) in luciferase reporter assay rs4143815*G allele was associated with a higher expression of PD-L1 due to disruption of the miR-570 binding site, hence this allele may be considered as potential cancer risk factor.

***rs2297136A>G*** is located in 3’UTR, in potential binding site for miR-296-5p (51) and miR-324-5p (51, 52). The study by Du et al. (51) showed that the expression in constructs containing rs2297136*G allele was significantly inhibited by miR-296-5p. From these data it can be concluded that rs2297136*G can be considered as protective allele in the context of cancer development, due to its association with decreased PD-L1 expression.

***rs10815225G>C*** is located in the promoter region in the SP1 consensus sequence. According to data presented by Tao et al. (49), SP1 bounds to the rs10815225*G allele with higher affinity than to the rs10815225*C allele. Moreover, *PD-L1* mRNA expression was higher in *rs10815225 G/G* homozygous gastric cancer patients in comparison to patients with the *rs10815225 G/C* genotype (49). Based on that, it can be inferred that rs10815225*C may be associated with lower cancer risk, due to the lower PD-L1 expression.

***rs4742098A>G*** is located in 3’UTR, in the miR binding site. According to literature data, the expression in constructs containing rs4742098*A allele was significantly suppressed by miR-138 (51). The rs4742098*A allele may be considered as protective in cancers overexpressing miR-138, due to promotion of lower PD-L1 expression. The possible functional relevance of *PD-L1* SNPs was summarized in **Table 2**.

### *PD-L1* Polymorphisms and Cancer Risk

#### Overall Cancer Risk

Polymorphisms in the *PD-L1* gene were not as broadly explored in the context of cancer as those in *PDCD1*. According to our best knowledge, two meta-analyses were conducted to estimate the potential association between *PD-L1* SNPs and overall cancer risk.

A meta-analysis by Zou et al. (190), including 11 articles (49–53, 173, 191–195) (3,711 cases, 3,704 controls), revealed association between the *rs4143815C>G* and overall susceptibility to cancer, with increased risk for rs4143815*G allele carriers (OR = 1.28). Similar results were obtained in a meta-analysis performed by Hashemi et al. (153). Additionally, Hashemi et al. (153) investigated the *rs2890658C>A*, however no evidence of association between this polymorphism and overall cancer risk was found.

#### Ovarian Cancer

According to our best knowledge, association between *PD-L1* SNPs and ovarian cancer was evaluated only in one study. Similarly, as in the case of overall cancer risk, increased susceptibility to ovarian cancer was observed for carriers of rs4143815*G allele (OR = 2.00) in the study performed on 164 patients and 170 control subjects (173). The carriers of rs4143815*G allele showed also higher differentiation grade.

#### Gastrointestinal Cancers

Stratified analysis performed as part of a meta-analysis by Hashemi et al. (153) (pooling gastrointestinal cancers together) revealed lower risk of gastrointestinal cancer for rs4143815*C allele carriers (OR = 0.64) (higher for *rs4143815 G/G* genotype), while no evidence of association was found for *rs2890658A>C* (153).

As far as particular types of gastrointestinal cancers are concerned, *PD-L1* SNPs have been investigated *inter alia* in esophageal squamous cell carcinoma. Zhou et al. examined two polymorphisms of *PD-L1*: *rs2890658A>C* and *rs4143815C>G* (195) (575 patients, 577 controls) and found association only for *rs2890658A>C* in smokers. In this subgroup the *rs2890658 A/C* genotype seemed to increase the risk of ESCC (OR = 1.51).

According to our best knowledge, in the literature there are also two articles that examined the potential associations between *PD-L1* SNPs and gastric cancer (49, 52). Both of them were included in the above mentioned subgroup analysis performed by Hashemi et al. (153). Of the two polymorphisms (*rs2297136A>G* and *rs4143815C>G*) examined by Wang et al. (52) on the group of 205 patients and 393 controls, only *rs4143815C>G* was reported as associated with susceptibility to GC, with the *rs4143815 G/G* genotype increasing risk (over 3.5-fold, OR = 3.73) (52). In an extended study carried out by the same research team (350 patients, 500 subjects) (49) the association of *rs4143815C>G* was confirmed, however this study revealed that the presence of one rs4143815*G allele was sufficient to cause an increased risk of gastric cancer (OR = 1.86). Moreover, in the study by Tao et al. (49) the association between *rs10815225G>C* and GC was observed. The presence of rs10815225*C allele decreased the risk of GC (OR = 0.60). It is worth noting that in this study was stated that both *rs4143815C>G* and *rs10815225G>C* were in HWE, however it can be calculated that for both of SNPs deviations from HWE exist in controls.

Also, in the case of HCC, the evaluation of potential associations between polymorphisms of *PD-L1* (*rs2297136A>G*, *rs4143815C>G*, rs2890658A>C, *rs17718883C>G*) and risk of developing this type of cancer was conducted (225 patients, 200 controls) (50). The results of this study indicated an association between *rs2297136 A/A* as well as *rs4143815 G/G* genotypes and increased risk of HCC (OR = 1.44 and OR = 1.62, respectively), while a decreased risk of HCC was observed for carriers of minor rs17718883*G allele (OR = 0.15). Since the frequency of alleles at *rs2297136A>G* polymorphic site in databases (Ensembl and dbSNP) for all populations (including Asian) is opposite to that presented in the article i.e., the minor allele at *rs2297136* is the *G* allele (not *A*), the results described by Xie et al. should be treated with caution.

An attempt to find evidence on a potential association between *PD-L1* genetic variations (*rs2890657G>C*, *rs822338T>C*, *rs10815225G>C*, *rs4143815C>G*, *rs866066C>T*) and another type of gastrointestinal cancer—colorectal cancer, was made by Catalano et al. (53) in the Czech population (1,424 CRC patients, 1,114 controls). Unfortunately, none of these polymorphisms was associated with risk of CRC in single SNP analysis. However, it is worth mentioning that the authors suggested the existence of interactions between *PD-L1* and *NLRC5* genes (53).

#### Non-Small Cell Lung Cancer

Polymorphisms of *PD-L1* gene were also examined in NSCLC. In a subgroup analysis by cancer type [being part of meta-analysis by Hashemi et al. (153)], including 3 articles (136, 196, 197), (1,109 cases and 1,193 controls), the association between *rs2890658A>C* and NSCLC was revealed. According to this analysis, carriers of the rs2890658*C allele possessed more than 1.5-fold higher susceptibility to NSCLC (OR = 1.77) in comparison to individuals with the *rs2890658 A/A* genotype (153). Such association was observed in each study included in the meta-analysis (136, 196, 197). Moreover, it was demonstrated by Ma et al. that NSCLC patients carrying rs2890658*C allele had increased risk of regional LN metastasis as compared to individuals with *rs2890658 A/A* genotype (OR = 5.65). However, all the aforementioned results concerning NSCLC should be treated cautiously since the frequency of alleles at *rs2890658* in databases (Ensembl and dbSNP) for all populations (including Asian) as well as in the study by Zhou et al. (for ESCC; describe below) is opposite to that presented in these articles i.e., the minor allele at *rs2890658* is A allele (not C).

Additionally, three SNPs in 3’UTR of *PD-L1* (*rs4143815C>G*, *rs2297136A>G*, and *rs4742098A>G*) were examined in NSCLC in the study performed on 320 patients and 199 control individuals by Du et al. (51). In the case of *2297136A>G* and *rs4742098A>G*, significant differences in genotype distribution between cases and controls were found. Increased risk of NSCLC was noted for *rs2297136 A/G* (OR = 2.29) as well as for *rs4742098 A/G* heterozygotes (OR = 1.60) (51). Furthermore, from this study it can be concluded that individuals with *rs2297136 G/G* genotype were less likely to exhibit LN metastasis (OR = 0.27) and more likely to exhibit distant metastasis (OR = 3.83) in comparison to subject with the *rs2297136 A/A* genotype. In case of *rs4742098A>G*, carriers of rs4742098*G allele had greater depth of tumor infiltration as compared to individuals with *rs4742098 A/A* (OR = 2.30). Taking into consideration the fact that this is the only study considering 3’UTR polymorphisms and NSCLC, further investigations are needed (especially since in this study deviations from HWE in control group existed for *rs2297136A>G* and *rs4143815C>G*, although in the manuscript is stated otherwise).

Although *rs4143815G>C* did not show the association with NSCLC risk and progression in the aforementioned study by Du et al. (51) it was demonstrated by Lee et al. (on 354 NSCLC patients) (135) that individuals with *rs4143815 G/G* genotype (G allele is a minor allele in Korean population) had shorter overall survival as compared to rs4143815*C allele carriers. Similar effect was also observed for two promoter polymorphisms of *PD-L1* gene—*rs822336G>C* and *rs822337T>A*. In details, subjects with *rs822336 C/C* as well as subjects with *rs822337 A/A* genotypes showed shorter overall survival as compared to rs822336*G and rs822337*T allele carriers, respectively. The above described associations between *PD-L1* polymorphisms and cancer risk are summarized in **Supplementary Table 3**.

## B- and T-Lymphocyte Attenuator

BTLA was discovered in 2003 by Gavrieli et al. (198) as a third co-inhibitory molecule being member of the CD28 family, similar to CTLA-4 and PD-1. In the same year, the first data regarding its expression and function were published (199). In 2005, BTLA’s ligand was identified. Surprisingly, in contrast to CTLA-4 and PD-1 ligands, the BTLA ligand - herpes virus entry mediator (HVEM) belongs to the TNF receptor (TNFR) superfamily (200). HVEM binds with many co-stimulatory and co-inhibitory molecules. The role of both types of co-signaling molecules are opposite, and are known as the “molecular switch” model of activation and inhibition. Crosslinking of LIGHT or LIGHT–α with HVEM provides a positive signal for lymphocyte proliferation, activation and inducing inflammatory reactions. Binding of HVEM to BTLA or CD160 exerts an adverse effect, and results with inhibition of T and B lymphocyte activation, proliferation and cytokine production (201).

**Figure 4:**
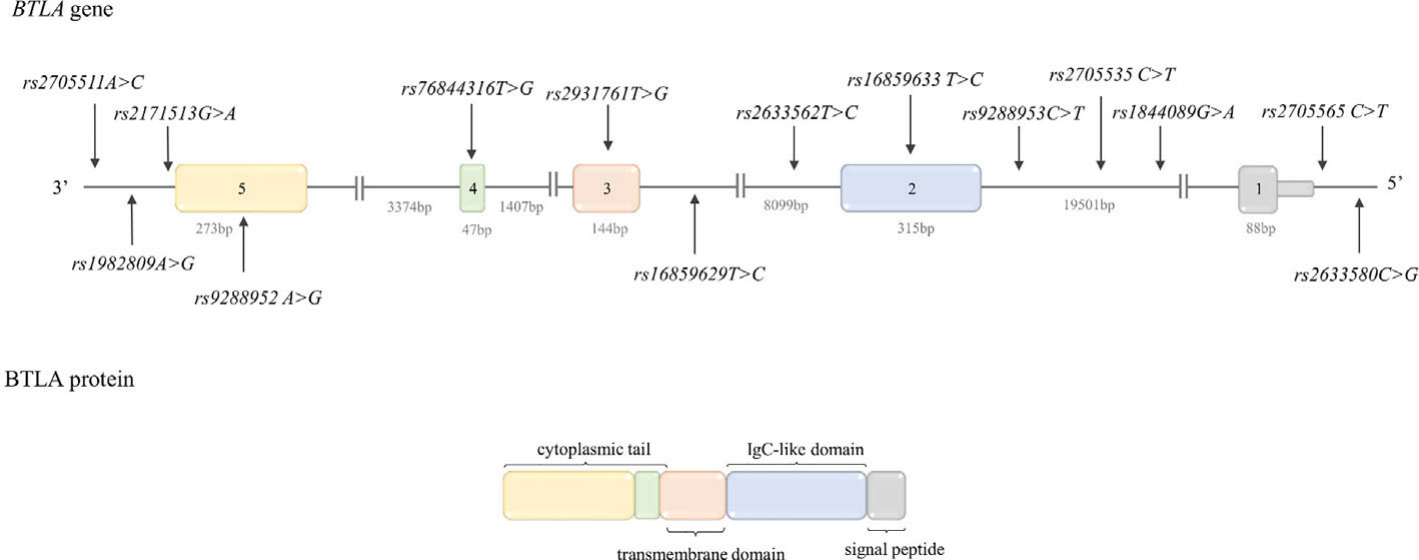
Structure of *BTLA* gene and protein. Top: *BTLA* gene structure. The figure shows the polymorphisms described in the review and the lengths of the exons and introns. Bottom: BTLA protein structure. The colors indicate which region of the protein is encoded by which exon.

Similarly, to CTLA-4 and PD-1, BTLA (also known as CD272) is a type 1 transmembrane glycoprotein comprising 289 amino acids (**Figure 4**) (199). BTLA differs from the CD28 family members (CD28, CTLA-4, ICOS, PD1) by having an extracellular C-like-domain, instead of a V-like-domain (148). On the cell surface, BTLA is presented as a monomer with a single IgC domain (148). It binds to the cysteine-rich domain (CRD)-1 of HVEM in stoichiometry 1:1 on the opposite face from the LIGHT (co-stimulatory receptor of HVEM) (200, 202).

BTLA is expressed almost exclusively on immune cells (199). BTLA expression on B cells is low in bone marrow during pro and pre B cell maturation, higher in immature B cells and high on resting peripheral B cells (203). Otsuki et al. (204) reported constitutive BTLA expression on the majority of CD4^+^ and CD8^+^ T cells, however upon T cell activation expression progressively decreased. In contrast to CTLA-4 and PD-1 which are highly expressed on T_reg_ cells, BTLA expression is weak, while it is highly expressed on anergic T cells generated by chronic exposure to antigens (205). Moreover, high expression of BTLA was observed on T follicular helper cells and DCs [reviewed in (203)].

Resting T cell inhibition by BTLA is stronger than the positive signal by HVEM which prevents the excessive activation of T cells (206). BTLA//HVEM binding inhibits antigen specific TCR signaling-mediated proliferation, activation (CD25, CD38) and production of cytokines: IL-2, IL-4, and IL-10. BTLA crosslink HVEM on T_reg_ cells facilitate their immunosuppressant effect. Similarly, to T and B lymphocytes, BTLA is expressed by NKT cells, and its expression inhibits cytokine (IL-2, IL4, and IFN-γ) secretion (207). Moreover, it was shown on mice models of breast cancer that blocking of BTLA pathway promotes the anticancer activity of NKT cells, which infiltrate tumors and inhibit tumor growth (208).

The *BTLA* gene is located on chromosome 3 in q13.2 in a reverse orientation. The *BTLA* gene structure is shown in **Figure 4**. *BTLA* has three splicing variants: full length BTLA has five exons and possess a Ig-C like domain; the BTLA isoform with 70% extracellular domain missing due to a skipped exon 3 and soluble BTLA which lacks a fragment of exon 4 due to alternative 3’ splice site caused by a premature stop codon (209).

### *BTLA* Gene Polymorphisms

As compared to *CTLA-4* and *PDCD1*, the *BTLA* gene polymorphisms has not been well studied yet. At the beginning in literature, there were only a few studies that addressed *BTLA* gene polymorphisms, and majority of them have investigated its role in susceptibility to autoimmune diseases, such as rheumatoid arthritis (RA) (55, 210), systemic lupus erythematosus (SLE) and type 1 diabetes mellitus (211). Below we described the most intensively examined genetic variants of *BTLA* in human diseases together with their functional relevance, if such data were available in literature.

The frequency of the described below *BTLA* genetic variants in different populations is presented in **Table 1**.

***rs1844089C>T* (G>A REV) and *rs2705535C>T* (G>A REV)** are located in first intron, the exact functional role of these SNPs was not established yet, however Ge at al. postulated that rs2705535 may affect splicing of *BTLA* gene (54).

***rs9288953C>T* (G>A REV)** is situated in the first intron. Ge et al. (54) postulate based on the human splicing finder software analysis, that rs9288953*T allele may potentially activate six new splice sites in splicing enhancer motifs and break one splicing sites in the silencer motif and in this way may perhaps promote higher BTLA expression (54).

***rs76844316T>G (BTLAc.590A>C* REV)** is located in exon 4, and leads to the exchange of asparagine to threonine at position 197 in the intracellular domain. Oki and colleagues (55) performed *in vitro* study in order to evaluate functional relevance of *BTLAc.590A>C* in Jurkat T cells transfected with construct with BTLAc.590*A or BTLAc.590*C allele. The transfected cells did not express different levels of surface BTLA. Stimulation of infected Jurkat cells with concanavalin A (ConA) indicated that IL-2 production was strongly inhibited in cells expressing BTLA with BTLAc.590*A, whereas in cells with BTLAc.590*C the IL-2 production was enhanced. Moreover, transfected cells were stimulated with anti-CD3 and anti-BTLA antibodies (Ab). Anti -BTLA Ab inhibited IL-2 production only in Jurkat expressing BTLAc.590*A. The authors suggest that BTLAc.590*C had not ability to inhibit IL-2 production by Jurkat cells.

Even though the amino acid exchange is not within the ITIM motifs of BTLA, it is hypothesized that this substitution may alter the posttranslational modifications of BTLA, such as glycosylation of asparagine, or phosphorylation of threonine by serine/threonine kinase, which may influence the strength of BTLA signaling by SHP1/SHP2 (212). Hence, BTLAc.590*A allele was associated with decreased inhibitory activity of BTLA in Jurkat cells it may potentially constitute cancer risk factor.

***rs9288952T>C (BTLAc.800G>A* REV)** is a non-synonymous SNP causing an amino acid substitution from proline to leucine at position 267 in exon 5, which was initially described as associated with susceptibility to RA (210).

***rs1982809T>C* (A>G REV)** is situated in 3’ nearby gene region of *BTLA* (73 bp). *In silico* analysis with application of SNPinfo (213) and FastSNP (214) databases did not provide data regarding potential biological implication of this SNP. However, it was shown that the presence of rs1982809*C allele was associated with lower mRNA expression level of *BTLA* in the subset of T cells of the CLL patients (56). Although, it can be postulated that BTLA rs1982809*T allele may confer increased susceptibility to cancer development, the published already data indicated that *C* allele confers susceptibility to several cancers (CLL, renal and lung cancer). Further studies are needed to established the functional role of this SNP.

***rs2705511A>C*** is located in the intragenic region between genes encoding CD200 and BTLA (-97820bp||**-**3334bp). *Rs2705511* and *rs1982809* are in moderate linkage disequilibrium with each other (215). In two databases SNPinfo (213) and FastSNP (214) there were no data about functional role of this polymorphism. The possible functional relevance of *BTLA* SNPs was summarized in **Table 2**.

### *BTLA* Gene Polymorphisms and Cancer Risk

#### Breast Cancer

Five SNPs of *BTLA* gene: *rs1844089C>T*, *rs2705535C>T*, *rs2633562T>C*, *rs2931761T>G*, and *rs9288952A>G* were investigated in Chinese women (592 patients, 506 controls) in relation to malignant BC risk (216). It was found that *rs1844089 C/T* and *rs2705535 C/T* genotypes increased the risk of BC 1.3 and 1.5 times respectively, while *rs1844089 C/C* genotype *rs2705535 C/C* genotype and *rs9288952 G/G* genotypes conferred 1.3, 1.4, and 1.7 times lower risk of BC, respectively. Moreover, the haplotype (*rs9288952, rs2931761, rs2633562, rs2705535, rs1844089)* GTTTT was associated with the three times increased risk of BC in this population. These authors analyzed also association between these SNPs and some clinical features. The frequency of *rs1844089 C/T* genotype was higher in patients with tumor size over 5 cm, while (*rs9288952, rs2931761, rs2633562, rs2705535, rs1844089*) *GTCTC* haplotype was significantly more frequent in patients with metastasis to LNs (216).

#### Gastrointestinal Cancers

Association between four *BTLA* SNPs (*rs16859629T>C*, *rs1982809A>G*, *rs2171513G>A*, and *rs3112270T>C*) and esophageal squamous cell carcinoma was investigated by Cao et al. (217) in 721 patients and 1,208 control subjects. None of the examined *BTLA* SNPs were associated with ESCC risk. However, it was shown that *T/C* genotype of *rs3112270T>C* slightly lowered (1.2 times) risk of ESCC development in males. In a stratified analysis by age, BMI, smoking status, and alcohol consumption it was noticed that *rs3112270 T/C* genotype may protect lean individuals with BMI<24 from ESCC (*T/C vs*. *T/T*; OR = 0.72). Opposite effect was observed for *rs3112270 C/C* genotype in subjects with BMI>24. In this group *C/C* genotype increased 2 times risk of ESCC (*C/C vs. T/T).* Additionally, the *G/A* genotype of *rs2171513G>A* (*G/A vs*. *A/A*) was associated with lower risk of ESCC (OR = 0.61) in individuals’, who overused alcohol (217).

Tang et al. examined relationship between *BTLA* SNPs (*rs16859629T>C*, *rs1982809A>G*, *rs2171513G>A*, and *rs3112270T>C*) and EGJA risk in 1,234 patients and 1,540 control subjects (218). None of these SNPs alone were associated with EGJA risk. However, haplotype analysis revealed that haplotype (*rs16859629, rs1982809, rs2171513, rs31122708*) *TAAG* increased three times risk of EGJA development. Moreover, after adjustment for gender, age, alcohol consumption and smoking, the *A/A* genotype of *rs1982809A>G* was associated with two times higher risk of EGJA in heavy smokers.

As mentioned previously Ge and coworkers (54) analyzed association between SNPs of genes encoding co-inhibitory molecules such as CTLA-4, PD-1 and BTLA and CRC risk (601 patients, 627 controls). Three SNPs within the *BTLA* gene were investigated: *rs1844089G>A*, *rs2705535C>T*, and *9288953C>T*. The *T/T* genotype of *rs2705535C>T* was associated with 2 times increased risk of rectal cancer development (in comparison to *C/T*+*C/C*), while the presence of *rs9288953 T/T* genotype decreased 1.4 times the risk of rectal cancer (in comparison to *C/T*+*C/C*) (54).

#### Hematological Malignancies

As far as potential association between *BTLA* polymorphisms and hematological malignancies is concerned, such evaluation was performed only for CLL. The association of *BTLA* gene polymorphisms and CLL risk were evaluated by Karabon et al. (56) in 321 patients and 470 control subjects. Among the investigated *BTLA* SNPs were: *rs2705511A>C*, *rs1982809A>G*, *rs9288952A>G*, *rs76844316T>G*, *rs16859633T>C*, *rs9288953C>T*, *rs2705535A>C*, *rs1844089G>A*, *rs2705565C>T*, and *rs2633580C>G*. The carriers of rs1982809*G allele and rs2705511*C allele were more prone to CLL development (OR = 1.5 and OR = 1.6, respectively). Additionally, the *T/T* genotype of *rs9288953C>T* was associated with 1.7 times higher risk of CLL.

#### Renal Cancer

Partyka et al. (219) carried out a case-control study on the group of 282 patients and 480 control subjects in order to evaluate association between the following *BTLA* SNPs *rs1844089G>A*, *rs2705535A>C*, *rs9288953C>T*, *rs9288952A>G*, *rs16859633T>C*, *rs1982809A>G*, *rs2705511A>C*, and renal cell carcinoma (RCC). The presence of rs1982809*G allele (*G/G* + *A/G*) was associated with 1.4 times higher risk of RCC, while *G/G* genotype was associated with higher risk (OR = 2.75) of the clear cell RCC (ccRCC) high grade tumors (219).

#### Lung Cancer

The association of the following genetic variants of *BTLA rs1982809A>G*, *rs9288952A>G* and *rs9288953C>T* with lung cancer risk was studied recently in the Tunisian population (196 patients, 300 controls) (220). The possession of rs1982809*G allele was associated with 1.5 increased risk of lung cancer development. The rs1982809*G allele was also associated with T4 tumor size (OR = 1.8), metastasis to LNs (OR = 3.71), and development of adenocarcinoma subtype of lung cancer (OR = 2.8) (220). The summary of above-described associations between *BTLA* polymorphisms and cancer risk is shown in **Supplementary Table 4**.

## T Cell Immunoglobulin and Mucin-Domain Containing-3

**Figure 5:**
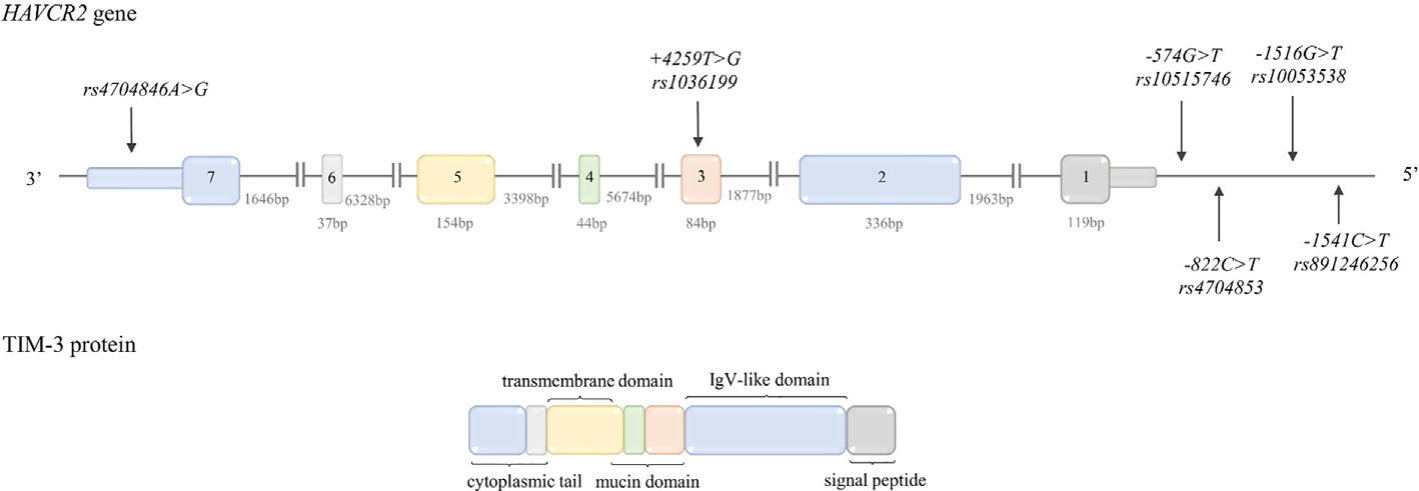
Structure of *HAVCR2 (TIM-3)* gene and protein. Top: *HAVCR2* gene structure. The figure shows the polymorphisms described in the review and the lengths of the exons and introns. Bottom: TIM-3 protein structure. The colors indicate which region of the protein is encoded by which exon.

T Cell Immunoglobulin And Mucin-Domain Containing-3 was discovered in 2002 (TIM-3) (59). TIM-3 also known as HAVCR2 (hepatitis A virus cellular receptor 2) belonging to the T cell transmembrane immunoglobulin and mucin domain (TIM) family is a type I transmembrane protein composed of 281 amino acids (**Figure 5**) (221). TIM-3 is an immune checkpoint receptor constitutively expressed on innate immune cells such as monocytes/macrophages, DCs, mast cells, and NK cells and on CD4^+^ (Th1), CD8^+^ (Tc1), and Th17 cells (222). TIM-3 expression by T cells is related to activated and terminally differentiated states.

TIM-3 is an important IC in terms of cancer, since it is highly expressed on TILs (Tumor Infiltrating Leukocytes). The PD-1^+^ TIM-3^+^ CD8^+^ T cells are considered to be the most dysfunctional T cells—a “deeply” exhausted T cell subset (222). CD8^+^ T cells with high expression of TIM-3 have been related to tumor progression (222). As mentioned earlier, TIM-3 is also expressed by NK cells and T_regs,_ and its important role in regulating functions of these cells in carcinogenesis has been recently demonstrated. TIM-3 is considered as a marker of exhausted NK cells and may be also responsible for their dysfunctional phenotype in cancer settings. Apart from suppressing immune response directly, TIM-3 is also able to inhibit it indirectly by fostering generation of myeloid-derived suppressor cells (MDSC) in a TIM-3/galectin-9 dependent manner (222). TIM-3 is also expressed by CD4^+^FoxP3^+^ T_reg_ cells and it has been demonstrated that it enhances the regulatory function of those cells (222).

Several ligands have been proposed for TIM-3: phosphatidyloserine (PtdSer) on apoptotic cells; high-mobility group box1 (HMGB-1) - a damage associated molecular pattern protein that is secreted by stressed innate immune cells; and galectin-9. It has been demonstrated that interaction between galectin-9 and TIM-3 resulted in inhibition of Th1 and Th17 response including peripheral tolerance (222, 223).

Human TIM-3 is encoded by the *HAVCR2* (minus strand) gene located on chromosome band 5q33.3 together with genes *HAVCR1* and *TIMD4* for other members of the TIM family, TIM-1 and TIM-4 respectively (222) and consists of seven exons encoding its full-length protein sequence comprising 301 aa. The *HAVCR2* gene structure is shown in **Figure 5**.

*In silico* analysis of 5’ upstream and the promoter region of *HAVCR2* gene predicted localization of putative binding sites for the TFs: GATA-1, YY-1, p300, HNF-3b, Pbx-1, RARa, NFE2, MZF-1, and GATA-3 (57). The T-bet and NFIL3 (Nuclear Factor, Interleukin 3 Regulated) TFs have been reported as being involved in the regulation of *HAVCR2* gene in T cells (224).

### *TIM-3/HAVCR2* Gene Polymorphisms

The frequency of the *HAVCR2* polymorphisms (described below) in different populations is presented in **Table 1**.

***rs891246256G>A* (*-1541C>T* REV)** and ***rs10053538C>A* (*-1516G>T* REV)** are the 2 kb upstream SNPs. The function for *rs891246256G>A* has not been established yet. The *rs10053538* is situated at the putative binding site of p300 TF (57). The presence of -1516*T allele (*G/T*+*T/T*) of this SNP has been shown to be associated with higher expression of TIM-3 on liver infiltrating lymphocytes in tumor tissue of HCC patients in comparison to *-1516 G/G* genotype as determined by immunohistochemistry (58)

***rs1036199A>C* (*+4259T>G* REV**) is located in exon 3. This is a missense polymorphism causing substitution from arginine to leucine in position 140 (R140L). The functional consequence of this change has not been reported so far but it can be postulated that this variant may affect the mucin domain (59). A meta-analysis aimed at evaluation of association between the *rs1036199* and autoimmune diseases (ADs) mainly in Asian populations pointed to the rs1036199*G allele as a risk factor increasing susceptibility to ADs (225). The possible functional relevance of *HAVCR* SNPs was summarized in **Table 2**.

### *TIM-3/HAVCR2* Gene Polymorphisms and Cancer Risk

#### Overall Cancer Risk

The *-1516G>T*, -*574G>T*, and *+4259T>G* of the *HAVCR2* gene are the most studied polymorphisms in terms of association with overall cancer risks. The published meta-analyses are related to Asian populations. In 2016, the first meta-analysis concerning these SNPs has been published by Gao et al. based on six published studies (178, 226–231) (2,039 cases, 2,372 controls) evaluating the association between *-1516G>T*, *-574T>G*, and +*4259T>G* and cancer risk in the Chinese Han population (232). This analysis revealed that the minor alleles in investigated SNPs: -1516*T (OR = 1.40), -574*T (OR = 1.99), and +4259*G (OR = 2.21) were associated with a higher overall cancer risk (232). The authors also analyzed association between those variants and cancer risk based on human systems. A potential association between higher risk of cancers of the digestive system was found for the -1516*T (789 cases, 992 controls) (OR = 1.79) and for the -574*T (722 cases, 856 controls) (OR = 1.77) alleles (232). The authors postulated that minor alleles of investigated variants may potentially cause higher individual risk of cancer by increasing TIM-3 expression or enhancing its function (232). Similar results were obtained in a meta-analysis performed by Fang et al. (233) based on eight studies (178, 226–228, 231, 234–236) (2,229 cases, 2,623 controls) concerning the Han Chinese population. Of note, five studies were also included in the report by Gao et al. (232). This meta-analysis took into consideration the following SNPs: *-1516G>T*, *-822C>T*, *-574G>T*, and *+4259 T>G*. Analysis for -*1516G>T* in terms of overall cancer risk was carried out for 2,229 cases and 2,623 controls. The pooled estimate suggested association between -1516*T allele and increased overall risk of developing cancer (OR = 1.33). This allele was also associated with digestive system cancer risk (OR = 1.61) (233) [based on three studies (228, 231, 234)]. As for *-822C>T* the authors did not make a pooled estimate due to only two available studies investigating this variant, however according to both of them (432 cases, 466 controls (231); 322 cases, 402 controls (234), the carriers of -822*T allele seemed to be more susceptible to the cancer development (OR = 2.21) (233). In the case of *-574G>T*, 2,074 cases and 2,385 controls were analyzed. The analyzed data showed that the -574*T allele might confer increased risk of overall cancer risk (OR = 2.39) (233). This study did not confirm association of allele -574*T with increased risk for the digestive system (233) which was described by Gao et al. (232). The association between the *+4259T>G* and overall cancer risk was assessed for 1868 cases and 2566 controls and it has been found that carriers of allele +4259*G possessed slightly higher risk of developing cancer in general (OR = 1.23). The authors concluded that their study clearly demonstrated involvement of *HAVCR2* gene polymorphisms (minor alleles of examined SNPs) in conferring higher risk for development of different human cancers (233).

#### Cancers of Digestive System

The association between *HAVCR2* genetic variants and the risk of gastric cancer (GC) development was published in 2010 by Cao at al (231). The authors investigated five SNPs: *-1541C>T*, *-1516 G>T*, *-822C>T*, *-574G>T*, and *+4259T>G* in 212 patients and 252 control subjects of the Chinese Han population. This study reported association between increased GC risk and the following genotypes: *-1516 G/T* (OR = 2.03), *-822 C/T* (OR = 3.19), and *-547 G/T* (OR = 2.74) (231). Additionally, the authors reported that the presence of -1516*T allele was associated with distant metastasis (OR = 2.21) in GC patients (231). Tong et al. (228) conducted a case-control study on 306 patients with pancreatic cancer (PC) and 422 control subjects of Han Chinese ethnicity in order to examine any possible association between *-1516G>T*, *-574G>T*, and *+4259T>G* and susceptibility to PC. The association was found only for *+4259T>G*, namely the carriers of the *+4259 T/G* genotype had almost three times higher risk (OR = 2.82) of PC development in comparison to subjects with the *G/G* genotype (228). To the best of our knowledge, so far only one study investigating an association between predisposition to colorectal cancer (CRC) development and SNPs of *HAVCR2* has been published. Zhang et al. (237) examined *-822C>T* and *+4259T>G* in a group of 258 patients and 246 control subjects from the Chinese population. The study revealed association between the *-882 T/T* (OR = 6.16) genotype and *+4259* G/G (OR = 5.11) and an increased risk of CRC (237).

#### Breast Cancer

The first study attempting to examine relations between the *HAVCR2* gene polymorphisms and susceptibility to breast cancer were carried out on 560 BC patients and 583 control subjects of Northwest China descent (238). The authors investigated the following *HAVCR2* SNPs: *-1516G>T*, *+4259T>G*, and *rs4704846A>G* and found that the carriers of the -1516*T allele had higher risk of BC development (OR = 1.37) in comparison to the G/G genotype. Among patients, possession of -1516*T allele was associated with increased risk of LN metastasis (OR = 1.68) (238). Additionally, immunohistochemical analysis demonstrated that *-1516 G/T* and *T/T* genotypes were associated with increased TIM-3 protein expression as compared to *G/G* genotype (238). In another published report Cheng et al. (239) assessed association between*-1516G>T*, -*574G>T*, *+4259T>G*, and risk of invasive BC in a group of 301 patients with invasive BC and 151 control individuals of Chinese Han ethnicity. The authors, unlike in the previously described publication, demonstrated that the *T/G* genotype of *+4259T>G* was associated with a higher risk of BC development (OR = 7.64) (of note *G/G* genotype was not detected in this study) as well as with the LN or distant metastasis in BC patients (OR = 3.16). The two remaining genetic variants were not associated with BC risk or clinical parameters (239).

#### Hematological Malignancies

As for hematological malignancies, the -*1516G>T*, *-574G>T*, and *+4259T>G* polymorphisms were investigated in non-Hodgkin lymphoma (NHL). A case-control study was performed on 496 NHL patients and 512 control subjects enrolled from the Han Chinese Population. The study demonstrated that subjects with the *-574 G/T* or *+4259 T/G* genotypes had more than two times higher risk for NHL development (OR = 2.72 and OR = 2.59, respectively) in comparison to subjects possessing wild type genotypes -*574 G/G* and *+4259 T/T* (226).

#### Non-Small Cell Lung Cancer

To the best of our knowledge so far only one report assessing the association of *HAVCR2* with NSCLC susceptibility has been published. Bai and colleagues (227) examined *-1516G>T*, *-574G>T*, and *+4259T>G* in 432 NSCLC patients and 466 control subjects from the Han Chinese population. The study revealed that *+4259 T/G* (*G/G* genotype was not determined neither in patients nor in the control group) genotype was associated with increased risk of NSCLC (OR = 2.81) (227). Moreover, patients carrying the *+4259 T/G* genotype had shorter survival in comparison to those with *T/T* genotype (15.2 *vs.* 27.7 months, respectively) (227).

#### Renal Cell Carcinoma

Cai and colleagues (230) investigated *-1516G>T*, *-574G>*T, and *+4259T>G* polymorphisms in 322 RRC patients and 402 control subjects of Chinese descent. Of the examined SNPs, association with increased RCC cancer risk was found for the *-574 G/T* (OR = 2.85) genotype and *+4259 T/G* genotype (OR = 3.34) (the -*574G>T T/T* and the *+4259 G/G* genotypes, were not determined in the investigated groups). Additionally, the +4259*G allele was associated in RCC patients with metastasis (OR = 2.18) (230). The summary of above presented associations between *HAVCR2* genetic variants and cancer risk is shown in **Supplementary Table 5**.

## Lymphocyte Activation Gene 3

**Figure 6:**
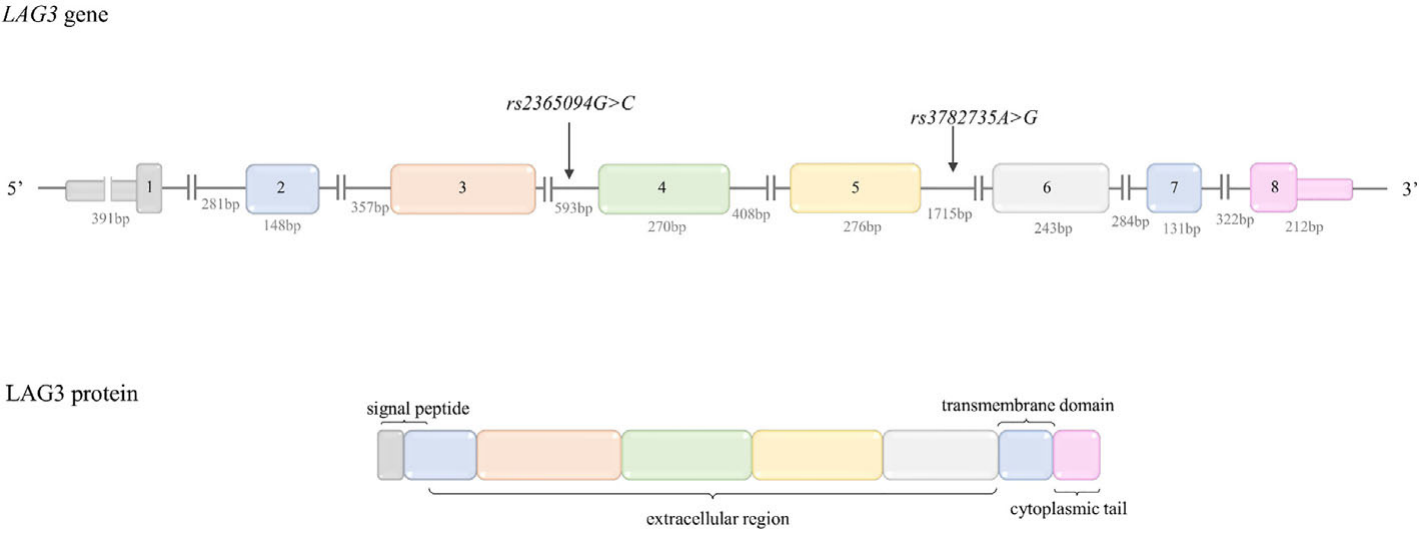
Structure of *LAG-3* gene and protein. Top: *LAG-3* gene structure. The figure shows the polymorphisms described in the review and the lengths of the exons and introns. Bottom: LAG-3 protein structure. The colors indicate which region of the protein is encoded by which exon.

Lymphocyte activation gene 3 is a heavily glycosylated type I transmembrane protein which shares significant homology with the CD4 molecule—in particular in its extracellular part, which consists of four immunoglobulin superfamily-like domains (D1-D4) (**Figure 6**).

LAG-3 is displayed on the surface of activated CD4^+^ and CD8^+^ T cells, B cells, NK cells, but also on plasmacytoid DCs (223). LAG3^+^ TILs have been identified in many tumor types, including lung, colon, breast, and pancreatic cancers and associated with aggressive clinical outcomes (240). LAG-3 is a negative regulator of CD4^+^ and CD8^+^ T cells. T_regs_ constitutively display the LAG-3 molecule and present increased suppressive activity. The LAG-3 expression on CD8^+^ TILs is associated with decreased proliferation rates and production of effector cytokines in cancer (241). A soluble LAG-3 (splice variant cleaved by metaloproteinases) is secreted in the cellular microenvironment and has immune-activating properties after binding to MHC-II expressed on APC (240). LAG-3 interacts with high affinity with its ligand—major histocompatibility complex class II (MHC II), Galectin 3 (soluble lectin expressed in a wide variety of cell types including tumor cells) and cell surface resident liver sinusoidal endothelial lectin (LSECtin, is expressed in the liver and on tumor cells) (223).

*LAG-3* gene was for the first time characterized by Triebel and colleagues in 1990 (242). The *LAG-3* gene is located on the 12p13.31 band, on the plus strand and spans about 5,952 bases. The *LAG-3* gene structure is shown in **Figure 6**.

### *LAG-3* Gene Polymorphisms

To the best of our knowledge, there is only one study available in the literature related to evaluation of association between cancer risk development and *LAG-3* genetic polymorphisms.

Lee and colleagues (243) performed a case-control study aimed at investigation of association between common SNPs in immunoregulatory genes and a risk of multiple myeloma in women. Genotyping was carried out in a group of 108 patients with MM and 482 control subjects. For two intronic variants of *LAG-3* gene *rs2365094G>C* and *rs3782735A>G* the potential association with MM risk was found. The carriers of rs2365094*C allele were more likely to develop (OR = 1.57) MM, whereas carriers of rs3782735*A allele were less likely to develop MM (OR = 0.69) (243).

The frequency of the *LAG-3* genetic variants (described below) in different populations is presented in **Table 1**.

## Conclusions and Future Perspectives

As was mentioned in the Introduction, the association between inherited variants in genes for ICs and cancer risk has been extensively investigated for CTLA-4 and broadly for PD-1/PD-L1 in a wide spectrum of cancers. Whereas, the potential targets for immune checkpoint blockers, like BTLA, TIM-3, and LAG-3 have been significantly less frequently investigated in such context. Moreover, there has been no publication to date that summarizes the knowledge on this subject. Therefore, our publication aims to fill this gap and collect currently available data and present the state of the art on that topic.

Our general impression derived from analysing of available literature survey is that SNPs may be considered as useful risk biomarkers in terms of cancer. From numerous studies it might be concluded that the CTLA-4c.49*A allele is associated with higher overall cancer risk alongside the risk of developing particular types of cancers, including breast, bone, and cervical cancers. Similarly, the CTLA-4c.-319*T increased an overall cancer risk in Caucasians, and predisposed to breast, cervical and hepatocellular cancers and bone cancer in Asian. The CTLA-4CT60*G allele was associated with higher risk of breast and hepatocellular cancers, while the CTLA-4c.-1661*G allele with overall, breast and gastric cancer risk.

As for *PDCD1* gene polymorphisms, the PD1.1*A allele seems to be associated with susceptibility to overall cancer in Asians as well as with susceptibility to ovarian, breast, and EGJA cancers (all studies in Asian populations). The available data suggest that the PD-1.5*T allele may protect from cancer development in general, and specifically against ovarian and breast cancers. The rs7421861*C allele increased overall cancer risk as well as the development of colorectal and EGJA cancers. Finally, it may be suggested that the PD-1.9*T allele may confer higher risk of ovarian and EGJA cancers development.

Only one polymorphism in the *PD-L1* gene, namely *rs4143815G>C* has been shown to be associated with cancer risk, in particular the rs4143815*G allele increased risk of overall, hepatocellular, ovarian, and gastric cancers.

Since the number of studies concerning *BTLA* gene polymorphisms and cancer risk is limited and since a meta-analysis of published results has not been performed yet, future research in this field is needed. On the basis of available literature, it can be however postulated, that rs1982809*G confer higher risk of lung cancer, CLL and renal cancer. The *rs9288953 G/G* genotype can be considered as a factor increasing risk of CLL and colorectal cancer, whereas rs1844089*T allele as a risk factor of breast cancer. Finally, rs2705535*A allele seems to be associated with higher risk of breast cancer and rs2705511*C with higher susceptibility to colorectal cancer and CLL.

**Figure 7:**
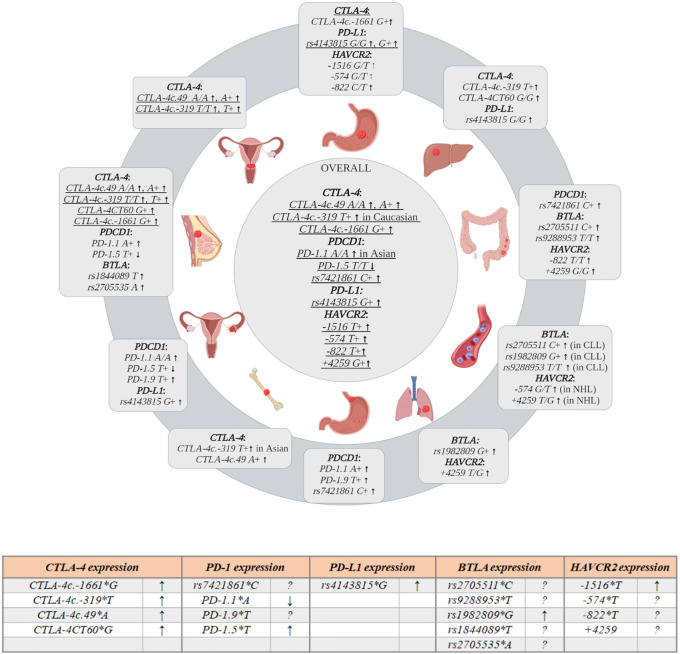
Graphical presentation of the association between inherited variations in genes encoding immune checkpoint (IC) molecules and risk of different types of cancers. Table presents association between polymorphisms and expression of ICs (↑↓ arrows indicate lower and higher expression, respectively; the question mark,?, no data on expression level are available). Figure was created with BioRender.com.

Similarly, to *BTLA* gene polymorphisms, genetic variants of the *HAVCR2* gene for TIM-3 have also been rarely investigated so far, however two meta-analyses have been published. According to them, the minor alleles of -*1516G>T*, -*822C>T*, *-574G>T*, and *+4259T>G* can be considered as overall cancer risk factors. In particular, the +4259*G allele was associated with the increased risk of lung cancer, colorectal cancer, and NHL; the -574*T allele with NHL and gastric cancer; -822*T allele with gastric cancer and colorectal cancer and -1516*T with gastric cancer. The associations between variants of genes encoding LAG-3 and TIGIT and cancer risk has not been evaluated yet and this area is almost unexplored. **Figure 7** summarized the main results of our review of literature.

A limitation of any conclusion arising from this literature survey is that most of the individual studies and meta-analysis were performed on Asian populations. It is worth mentioning that significant differences in distribution of genotypes between populations are observed and some of the presented associations are true only for individuals of Asian origin (**Table 1**).

As association between particular ICs’ SNPs and cancer risk seems to be relevant to specific population there is the urgent need to perform well designed studies on large scale in different populations in order to elucidate variants important in terms of risk estimation and selection of patients to the immunotherapy for particular ethnicities.

The next issue concerns the quality of some studies, as well as numbers of patients and controls in compared cohorts. We observed the following negligence in carrying out case-control studies and meta-analyses which may influence the reliability of published data. In several reports we noted deviation from HWE in control subject which was not mentioned, and even worst the authors stated that such deviation was not observed. We came across a meta-analysis which was published in the same form in another journal. Another issue which created considerable difficulties in proper interpretation of analyzed data was serious inconsistency in describing SNPs and their alleles. The authors use the old, common names of SNPs without providing SNP reference numbers. Some of analyzed genes are located in minus orientation and we noticed that authors do not pay attention to that describing alleles in relation to plus or minus strand even within one work. This is particularly valid for SNPs causing G to C or C to G substitution if their frequency is similar in the population (~50% for both alleles).

As a conclusion from this study, we propose future directions of the research broadening and complementing presented topic.

We think that in the future the researchers should analyze SNPs which will cover the whole genes of interest. The analysis of constellation of SNPs will allow to evaluate the phenotypic effect of particular haplotypes on immune cells which may allow evaluate if particular haplotype is associated with higher, lower or neutral risk of cancer development (34). Moreover, examination of potential interactions between SNPs, particularly in the context of receptor-ligand interaction may provide new interesting data of clinical significance.

It is also worth to analyze the impact of variation within genes encoding IC in context of tumor progression (among others impact on metastasis and overall survival).

Very important, but relatively poorly investigated is functional relevance of inherited variations not only in context of gene transcription, but also on gene translation. Little is also known about influence of SNPs on epigenetic gene regulation.

TIGIT is considered as a new target for immunotherapy due to its important role in maintenance of immunosuppressive phenotype of T cells in TM. Nonetheless, to the best of our knowledge, in the literature there is no data concerning TIGIT polymorphisms in relation to cancer risk. The future research will need to fill this gap in our knowledge.

In conclusion, the variants in genes encoding the molecules which regulate the immune surveillance might be considered as low-risk variants (OR<2) for cancer development, which has been well documented by numerous reports for *CTLA-4, PDCD1, PD-L1* genes, while more studies are needed for *BTLA, TIM3, LAG3*, and *TIGIT*.

## Conflict of Interest

The authors declare that the research was conducted in the absence of any commercial or financial relationships that could be construed as a potential conflict of interest.

## Authors Contributions

The authors participated equally in conception, writing, and revising the manuscript. MW prepared the figures. All authors contributed to the article and approved the submitted version.

## Funding

This work was supported by the National Science Centre, Poland, grant number 2019/33/B/NZ5/03029, as well as by the Hirszfeld Institute of Immunology and Experimental Therapy statutory program number 28/2020.
