# Supplementary material for: Immune Checkpoint Molecules—Inherited Variations as Markers for Cancer Risk
Source: Front Immunol. 2021 Jan 14;11:606721. doi: 10.3389/fimmu.2020.606721 (PMC7840570; doi:10.3389/fimmu.2020.606721)
Supplement: Supplementary file 1 [file Table_1.docx]

| **Cancer** | ***CTLA-4* polymorphisms** | | | | | |
| --- | --- | --- | --- | --- | --- | --- |
|  | ***c.49A>G***  **(rs231775)** | ***c.-319C>T***  **(rs5742909)** | ***CT60***  **(rs3087243)** | ***c. -1661A>*G**  **(rs4553808)** | ***c. -1722 T>C***  **(rs733618)** | ***c.-658 C>T*  (rs11571317)** |
| **Overall cancer risk** | AA↑[21;22]  A+↑[18] | T+↑[22] | No association [8] | G+↑[23] | No association [5] | No data |
| **Cervical cancer** | AA↑[1-3]  A+↑ | TT↑[4]^a^  T+↑ | Inconsistent results | No data | No data[5] | No data |
| **Breast cancer** | AA↑[6;7]  A+↑ | TT↑[7]  T+↑ | G+↑[6-8] | G+↑ [6;7] | No association [5] | T+↑[9] |
| **Gastric cancer** | No association [10] | C+↑ [10] | No association [10] | AA or AG↑[10] | No association [5;10] | No association [10] |
| **Colorectal cancer** | Inconsistent results[10-13] | No association [10] | No association [10] | No association [10] | No association [5;10] | No data |
| **Hepatocellular cancer** | Inconsistent results AA↑ [14]  No association [10] | T+↑[14] | GG↑[10] | No data | No data | No data |
| **Bone cancer** | A+↑[15;16] | T+↑[15] | no[16] | no[16] | No data | No data |
| **Hematological malignances** | No [17] | T+↑[17]^b^ | No [17] | No [17] | No data | No data |
| **Lung cancer** | Inconsistent results  A+↑[18] | No association [19;20] | No association [19;20] | No association [19;20] | No association [19;20] | No data |

Supplementary Table 1. Summary of results concerning associations between *CTLA-4* polymorphisms and risk of different types of cancers.

^a^ in Asian

^b^ trend in Caucasian

^c^ in Caucasian

Reference List

1 Liu P, Xu L, Sun Y, Wang Z: The association between cytotoxic T lymphocyte-associated antigen-4 and cervical cancer. Tumour Biol 2014;35:2893-2903.

2 Qiu H, Tang W, Yin P, Cheng F, Wang L: Cytotoxic Tlymphocyte-associated antigen4 polymorphisms and susceptibility to cervical cancer: a metaanalysis. Mol Med Rep 2013;8:1785-1794.

3 Zhang X, Zhang L, Tian C, Yang L, Wang Z: Genetic variants and risk of cervical cancer: epidemiological evidence, meta-analysis and research review. BJOG 2014;121:664-674.

4 Hu S, Pu D, Xia X, Guo B, Zhang C: CTLA-4 rs5742909 polymorphism and cervical cancer risk: A meta-analysis. Medicine (Baltimore) 2020;99:e19433.

5 Tang W, Qiu H, Jiang H, Sun B, Wang L, Yin J, Gu H: Lack of association between cytotoxic T-lymphocyte antigen 4 (CTLA-4) -1722T/C (rs733618) polymorphism and cancer risk: from a case-control study to a meta-analysis. PLoS One 2014;9:e94039.

6 Dai Z, Tian T, Wang M, Liu X, Lin S, Yang P, Liu K, Zheng Y, Xu P, Liu M, Yang X, Dai Z: CTLA-4 polymorphisms associate with breast cancer susceptibility in Asians: a meta-analysis. PeerJ 2017;5:e2815.

7 Chen H, Qi X, Bai X, Qiu P, Chen B: Role of CD152 genetic polymorphisms in the susceptibility to breast cancer. Oncotarget 2017;8:26679-26686.

8 Zhao HY, Duan HX, Gu Y: Meta-analysis of the cytotoxic T-lymphocyte antigen 4 gene +6230G/A polymorphism and cancer risk. Clin Transl Oncol 2014;16:879-885.

9 Goske M, Ramachander VRV, Komaravalli PL, Rahman PF, Rao C, Jahan P: CTLA-4 Genetic Variants (rs11571317 and rs3087243): Role in Susceptibility and Progression of Breast Cancer. World J Oncol 2017;8:162-170.

10 Li J, Wang W, Sun Y, Zhu Y: CTLA-4 polymorphisms and predisposition to digestive system malignancies: a meta-analysis of 31 published studies. World J Surg Oncol 2020;18:55.

11 Jiang B: Current Evidence on the Association between Cytotoxic T-Lymphocyte Antigen 4 +49G > A Polymorphism and Digestive System Cancer Risks: a Meta-analysis Involving 11,923 Subjects; 2013.

12 Wang Y, Wang X, Zhao R: The association of CTLA-4 A49G polymorphism with colorectal cancer risk in a Chinese Han population. Int J Immunogenet 2015;42:93-99.

13 Zhang Y, Zhang S, Xia W, Dong Z: Association of cytotoxic T-lymphocyte antigen 4 rs231775 gene polymorphism with colorectal cancer risk. J Cancer Res Ther 2018;14:S526-S532.

14 Wang C, Liu W, Zhao L, Dong Z: Association of cytotoxic T-lymphocyte antigen-4 + 49A/G gene polymorphism with hepatocellular carcinoma risk in Chinese. J Cancer Res Ther 2018;14:S1117-S1120.

15 Wang X, Liu Z: Systematic meta-analysis of genetic variants associated with osteosarcoma susceptibility. Medicine (Baltimore) 2018;97:e12525.

16 Bilbao-Aldaiturriaga N, Patino-Garcia A, Martin-Guerrero I, Garcia-Orad A: Cytotoxic T lymphocyte-associated antigen 4 rs231775 polymorphism and osteosarcoma. Neoplasma 2017;64:299-304.

17 Dai Z, Feng C, Zhang W, Liu J, Cao X, Zhang H, Liu Y, Wang M, Liu X, Dai Z: Lack of association between cytotoxic T-lymphocyte antigen-4 gene polymorphisms and lymphoid malignancy risk: evidence from a meta-analysis. Ann Hematol 2016;95:1685-1694.

18 Geng R, Song F, Yang X, Sun P, Hu J, Zhu C, Zhu B, Fan W: Association between cytotoxic T lymphocyte antigen-4 +49A/G, -1722T/C, and -1661A/G polymorphisms and cancer risk: a meta-analysis. Tumour Biol 2014;35:3627-3639.

19 Lee SY, Jung DK, Choi JE, Jin CC, Hong MJ, Do SK, Kang HG, Lee WK, Seok Y, Lee EB, Jeong JY, Shin KM, Yoo SS, Lee J, Cha SI, Kim CH, Park JY: Functional polymorphisms in PD-L1 gene are associated with the prognosis of patients with early stage non-small cell lung cancer. Gene 2017;599:28-35.

20 Khaghanzadeh N, Erfani N, Ghayumi MA, Ghaderi A: CTLA4 gene variations and haplotypes in patients with lung cancer. Cancer Genet Cytogenet 2010;196:171-174.

21 Sun T, Zhou Y, Yang M, Hu Z, Tan W, Han X, Shi Y, Yao J, Guo Y, Yu D, Tian T, Zhou X, Shen H, Lin D: Functional genetic variations in cytotoxic T-lymphocyte antigen 4 and susceptibility to multiple types of cancer. Cancer Res 2008;68:7025-7034.

22 Zhang Y, Zhang J, Deng Y, Tian C, Li X, Huang J, Fan H: Polymorphisms in the cytotoxic T-lymphocyte antigen 4 gene and cancer risk: A meta-analysis. Cancer 2011.

23 Yan Q, Chen P, Lu A, Zhao P, Gu A: Association between CTLA-4 60G/A and -1661A/G polymorphisms and the risk of cancers: a meta-analysis. PLoS One 2013;8:e83710.
