# Supplementary material for: Immune Checkpoint Molecules—Inherited Variations as Markers for Cancer Risk
Source: Front Immunol. 2021 Jan 14;11:606721. doi: 10.3389/fimmu.2020.606721 (PMC7840570; doi:10.3389/fimmu.2020.606721)
Supplement: Supplementary file 2 [file Table_2.docx]

Supplementary Table 2. Summary of results concerning associations between *PDCD1* polymorphisms and risk of different types of cancers.

| **Cancer** | ***PD1* polymorphisms** | | | | | | |
| --- | --- | --- | --- | --- | --- | --- | --- |
|  | ***PD-1.1 rs36084323G>A*** | ***PD-1.3 rs11568821G>A*** | ***PD-1.5***  ***rs2227981C>T*** | ***PD-1.6 rs10204525A>G*** | ***PD-1.9 rs2227982C>T*** | ***rs7421861T>C*** | ***rs41386349C>T*** |
| **Overall cancer risk** | AA↑ in Asian [1] | A+↓ [2]  GA ↓ [3, 4] | TT↓ [2-5] | no association | no association | C+↑ [2] | no data |
| **Breast cancer** | A+↑ [6] | no association | Inconsistent results  (T+↓ [2, 5, 6];  no association [7]) | no association | Inconsistent results (no association [6];  T+↓ [2, 8]) | no association | no data |
| **Cervical cancer** | no data | no data | TT↓ [9]  CT ↑ [10]  no association [11] | no data | no data | no data | no data |
| **Ovarian cancer** | AA↑ [12] | no data | T+↓ [12] | no data | T+↑ [13] | no data | no data |
| **Lung cancer** | no association | no association | Inconsistent results  (T+↓ [2];  no association [14, 15]) | no data | no association | no data | no data |
| **Gastrointestinal cancer** | no data | no data | TT↓ [2] | no data | T+↑ [2] | C+↑ [2] | no data |
| **Esophageal squamous cell carcinoma** | no association | no data | no data | Inconsistent results (GG↓ [16, 17];  G+↑ [18]) | T+↑ in females [17] | Inconsistent results (CC↓ [18];  no association [16] | no data |
| **Esophagogastric junction adenocarcinoma** | A+↑ [19] | no data | no data | no association | T+↑ [19] | C+↑[19] | no data |
| **Gastric cancer** | no data | no data | T+↑ [20] | no association | T+↑ [21] among ever drinking cases | no association | no data |
| **Colorectal cancer** | A+↓ [22]only in colon cancer | AA↑ [23] | CT↑ [24] only in colon cancer | no association | no association | C+↑ [25] | no data |
| **Hepatocellular cancer** | no data | no association | no data | G↓ [26] | no data | no data | no data |
| **Hematological malignances** | no association | no association | no association | no data | no association  (CT↓ [27]in leukemia) | no data | no association |
| **Thyroid cancer** | no data | no association | T+↑ [28] | no data | no data | no data | no data |
| **Head and Neck Squamous Cell Carcinomas** | no data | no association | no association | no data | no association | no data | no data |
| **Brain tumor** | no data | no association | T+↑ [29] | no data | no data | no data | no association |
| **Cutaneous melanoma** | no association | no data | no association | no data | no association | no data | no data |
| **Basal cell carcinoma** | no data | A+↓ [30] | no association | no data | no data | no data | no data |

1. Da, L.S., et al., *The PD-1 rs36084323 A > G polymorphism decrease cancer risk in Asian: A meta-analysis.* Pathol Res Pract, 2018. **214**(11): p. 1758-1764.

2. Hashemi, M., et al., *Association between PD-1 and PD-L1 Polymorphisms and the Risk of Cancer: A Meta-Analysis of Case-Control Studies.* Cancers (Basel), 2019. **11**(8).

3. Zhang, J., et al., *The association between polymorphisms in the PDCD1 gene and the risk of cancer: A PRISMA-compliant meta-analysis.* Medicine (Baltimore), 2016. **95**(40): p. e4423.

4. Dong, W., et al., *Programmed Cell Death-1 Polymorphisms Decrease the Cancer Risk: A Meta-Analysis Involving Twelve Case-Control Studies.* PLoS One, 2016. **11**(3): p. e0152448.

5. Tang, W., et al., *Programmed death-1 (PD-1) rs2227981 C > T polymorphism is associated with cancer susceptibility: a meta-analysis.* Int J Clin Exp Med, 2015. **8**(12): p. 22278-85.

6. Hua, Z., et al., *PD-1 polymorphisms are associated with sporadic breast cancer in Chinese Han population of Northeast China.* Breast Cancer Res Treat, 2011. **129**(1): p. 195-201.

7. Haghshenas, M.R., et al., *Program death 1 (PD1) haplotyping in patients with breast carcinoma.* Mol Biol Rep, 2011. **38**(6): p. 4205-10.

8. Ren, H.T., et al., *PD-1 rs2227982 Polymorphism Is Associated With the Decreased Risk of Breast Cancer in Northwest Chinese Women: A Hospital-Based Observational Study.* Medicine (Baltimore), 2016. **95**(21): p. e3760.

9. Ivansson, E.L., I. Juko-Pecirep, and U.B. Gyllensten, *Interaction of immunological genes on chromosome 2q33 and IFNG in susceptibility to cervical cancer.* Gynecol Oncol, 2010. **116**(3): p. 544-8.

10. Li, X.F., et al., *Association of the programmed cell death-1 PD1.5 C>T polymorphism with cervical cancer risk in a Chinese population.* Genet Mol Res, 2016. **15**(1).

11. Guzman, V.B., et al., *New approach reveals CD28 and IFNG gene interaction in the susceptibility to cervical cancer.* Hum Mol Genet, 2008. **17**(12): p. 1838-44.

12. Li, Y., et al., *The effect of polymorphisms in PD-1 gene on the risk of epithelial ovarian cancer and patients' outcomes.* Gynecol Oncol, 2017. **144**(1): p. 140-145.

13. Tan, D., L. Sheng, and Q.H. Yi, *Correlation of PD-1/PD-L1 polymorphisms and expressions with clinicopathologic features and prognosis of ovarian cancer.* Cancer Biomark, 2018. **21**(2): p. 287-297.

14. Ma, Y., et al., *Polymorphisms of co-inhibitory molecules (CTLA-4/PD-1/PD-L1) and the risk of non-small cell lung cancer in a Chinese population.* Int J Clin Exp Med, 2015. **8**(9): p. 16585-91.

15. Pirdelkhosh, Z., et al., *Investigation of Programmed Cell Death-1 (PD-1) Gene Variations at Positions PD1.3 and PD1.5 in Iranian Patients with Non-small Cell Lung Cancer.* Middle East J. Cancer, 2018. **9**: p. 5.

16. Qiu, H., et al., *Programmed death-1 (PD-1) polymorphisms in Chinese patients with esophageal cancer.* Clin Biochem, 2014. **47**(7-8): p. 612-7.

17. Zhou, R.M., et al., *Association of programmed death-1 polymorphisms with the risk and prognosis of esophageal squamous cell carcinoma.* Cancer Genet, 2016. **209**(9): p. 365-375.

18. Zang, B., C. Chen, and J.Q. Zhao, *PD-1 gene rs10204525 and rs7421861 polymorphisms are associated with increased risk and clinical features of esophageal cancer in a Chinese Han population.* Aging (Albany NY), 2020. **12**(4): p. 3771-3790.

19. Tang, W., et al., *Programmed death-1 polymorphisms is associated with risk of esophagogastric junction adenocarcinoma in the Chinese Han population: A case-control study involving 2,740 subjects.* Oncotarget, 2017. **8**(24): p. 39198-39208.

20. Savabkar, S., et al., *Programmed death-1 gene polymorphism (PD-1.5 C/T) is associated with gastric cancer.* Gastroenterol Hepatol Bed Bench, 2013. **6**(4): p. 178-82.

21. Tang, W., et al., *Programmed death-1 (PD-1) polymorphism is associated with gastric cardia adenocarcinoma.* Int J Clin Exp Med, 2015. **8**(5): p. 8086-93.

22. Shamsdin, S.A., et al., *Associations of ICOS and PD.1 Gene Variants with Colon Cancer Risk in The Iranian Population.* Asian Pac J Cancer Prev, 2018. **19**(3): p. 693-698.

23. Yousefi, A.R., et al., *PD-1Gene Polymorphisms in Iranian Patients With Colorectal Cancer.* Laboratory Medicine, 2013. **44**(3): p. 241-244.

24. Mojtahedi, Z., et al., *Programmed death-1 gene polymorphism (PD-1.5 C/T) is associated with colon cancer.* Gene, 2012. **508**(2): p. 229-32.

25. Ge, J., et al., *Association between co-inhibitory molecule gene tagging single nucleotide polymorphisms and the risk of colorectal cancer in Chinese.* J Cancer Res Clin Oncol, 2015. **141**(9): p. 1533-44.

26. Li, Z., et al., *Genetic variations of PD1 and TIM3 are differentially and interactively associated with the development of cirrhosis and HCC in patients with chronic HBV infection.* Infect Genet Evol, 2013. **14**: p. 240-6.

27. Ramzi, M., et al., *Genetic Variation of Costimulatory Molecules, Including Cytotoxic T-Lymphocyte Antigen 4, Inducible T-Cell Costimulator, Cluster Differentiation 28, and Programmed Cell Death 1 Genes, in Iranian Patients With Leukemia.* Exp Clin Transplant, 2018.

28. Haghshenas, M.R., et al., *Association of PDCD1 gene markers with susceptibility to thyroid cancer.* J Endocrinol Invest, 2017. **40**(5): p. 481-486.

29. Namavar Jahromi, F., et al., *Association of PD-1.5 C/T, but Not PD-1.3 G/A, with Malignant and Benign Brain Tumors in Iranian Patients.* Immunol Invest, 2017. **46**(5): p. 469-480.

30. Fathi, F., et al., *Association of programmed death-1 gene polymorphisms with the risk of basal cell carcinoma.* Int J Immunogenet, 2019. **46**(6): p. 444-450.
