# Supplementary material for: Immune Checkpoint Molecules—Inherited Variations as Markers for Cancer Risk
Source: Front Immunol. 2021 Jan 14;11:606721. doi: 10.3389/fimmu.2020.606721 (PMC7840570; doi:10.3389/fimmu.2020.606721)
Supplement: Supplementary file 3 [file Table_3.docx]

| **Cancer** | ***PD-L1* polymorphisms** | | | | | | | |
| --- | --- | --- | --- | --- | --- | --- | --- | --- |
|  | ***rs4143815C>G*** | ***rs2297136A>G*** | ***rs10815225C>G*** | ***rs4742098A>G*** | ***rs17718883C>G*** | ***rs2890657G>C*** | ***rs2890658C>A*** | ***rs822338T>C*** |
| **Overall cancer risk** | G+↑ [1, 2] | no data | no data | no data | no data | no data | no data | no association |
| **Lung cancer** | no association | AG↑ [3] | no data | AG↑ [3] | no data | no data | C+↑ [2, 4-6] | no data |
| **Ovarian cancer** | G+↑ [7] | no data | no data | no data | no data | no data | no data | no data |
| **Gastrointestinal cancer** | GG↑ [2] | no data | no data | no data | no data | no data | no data | no association |
| **Gastric cancer** | GG↑ [8]  G+↑ [9] | no association | C+↓ [9] | no data | no data | no data | no data | no data |
| **Esophageal squamous cell carcinoma** | no association | no data | no data | no data | no data | no data | AC↑ in smokers [10] | no data |
| **Colorectal cancer** | no association [11] | no data | no association [11] | no data | no data | no association [11] | no data | no association [11] |
| **Hepatocellular cancer** | GG↑ [12] | AA↑ [12] | no data | no data | G+↓ | no data | no association | no data |

Supplementary Table 3. Summary of results concerning associations between *PD-L1* polymorphisms and risk of different types of cancers.

1. Zou, J., et al., *Association of PD-L1 gene rs4143815 C>G polymorphism and human cancer susceptibility: A systematic review and meta-analysis.* Pathol Res Pract, 2019. **215**(2): p. 229-234.

2. Hashemi, M., et al., *Association between PD-1 and PD-L1 Polymorphisms and the Risk of Cancer: A Meta-Analysis of Case-Control Studies.* Cancers (Basel), 2019. **11**(8).

3. Du, W., et al., *Variant SNPs at the microRNA complementary site in the B7H1 3'untranslated region increase the risk of nonsmall cell lung cancer.* Mol Med Rep, 2017. **16**(3): p. 2682-2690.

4. Chen, Y.B., et al., *Association between single nucleotide polymorphism of PD-L1 gene and non-small cell lung cancer susceptibility in a Chinese population.* Asia Pac J Clin Oncol, 2014. **10**(2): p. e1-6.

5. Cheng, S., et al., *PD-L1 gene polymorphism and high level of plasma soluble PD-L1 protein may be associated with non-small cell lung cancer.* Int J Biol Markers, 2015. **30**(4): p. e364-8.

6. Ma, Y., et al., *Polymorphisms of co-inhibitory molecules (CTLA-4/PD-1/PD-L1) and the risk of non-small cell lung cancer in a Chinese population.* Int J Clin Exp Med, 2015. **8**(9): p. 16585-91.

7. Tan, D., L. Sheng, and Q.H. Yi, *Correlation of PD-1/PD-L1 polymorphisms and expressions with clinicopathologic features and prognosis of ovarian cancer.* Cancer Biomark, 2018. **21**(2): p. 287-297.

8. Wang, W., et al., *A miR-570 binding site polymorphism in the B7-H1 gene is associated with the risk of gastric adenocarcinoma.* Hum Genet, 2013. **132**(6): p. 641-8.

9. Tao, L.H., et al., *A polymorphism in the promoter region of PD-L1 serves as a binding-site for SP1 and is associated with PD-L1 overexpression and increased occurrence of gastric cancer.* Cancer Immunol Immunother, 2017. **66**(3): p. 309-318.

10. Zhou, R.M., et al., *Programmed death-1 ligand-1 gene rs2890658 polymorphism associated with the risk of esophageal squamous cell carcinoma in smokers.* Cancer Biomark, 2017. **21**(1): p. 65-71.

11. Catalano, C., et al., *Investigation of single and synergic effects of NLRC5 and PD-L1 variants on the risk of colorectal cancer.* PLoS One, 2018. **13**(2): p. e0192385.

12. Xie, Q., et al., *Correlations of PD-L1 gene polymorphisms with susceptibility and prognosis in hepatocellular carcinoma in a Chinese Han population.* Gene, 2018. **674**: p. 188-194.
