# Supplementary material for: Immune Checkpoint Molecules—Inherited Variations as Markers for Cancer Risk
Source: Front Immunol. 2021 Jan 14;11:606721. doi: 10.3389/fimmu.2020.606721 (PMC7840570; doi:10.3389/fimmu.2020.606721)
Supplement: Supplementary file 4 [file Table_4.docx]

Supplementary Table 4. Summary of results concerning associations between *BTLA* polymorphisms and risk of different types of cancers.

| **Cancer** | ***BTLA* polymorphisms** | | | | | | | | |
| --- | --- | --- | --- | --- | --- | --- | --- | --- | --- |
|  | ***rs2705511*** | ***rs1982809*** | ***BTLAc.800G>A (rs9288952)*** | ***rs16859633*** | ***rs9288953*** | ***rs2705535*** | ***rs1844089*** | ***rs2705565*** | ***rs312270*** |
| **Breast cancer** [1] | no data | no data | CC↓ | no data | no data | AG↑ GG↓ | CT↑ CC↓ | no data | no data |
| **Esophageal squamous cell carcinoma** [2] | no data | no associations | no data | no data | no data | no data | no data | no data | AG↓ in males, and in BM1<24  AG↑ in BM1>24 |
| **Esophagogastric junction adenocarcinoma** [3] | no data | AA↑ for ever smokers | no data | no data | no data | no data | no data | no data | no association |
| **Colorectal cancer** [4] | no data | no data | no data | no data | TT↓ in rectal cancer | TT↑ in rectal cancer | ? | no data | no data |
| **Hematological malignancies** [5] | C+↑ in CLL | G+↑ in CLL | no association | no association | TT↑ in CLL | no association | no association | no association | no data |
| **Renal cancer** [6] | no association | G+↑ | no association | no association | no association | no association | no association | no data | no data |
| **Lung cancer** [7] |  | G+↑ | no association |  | no association | no data | no data | no data | no data |

rs2705565 rs2633580 no association with hematological malignancies

rs2931761 rs2633562 no association with breast cancer

Rs16859629 rs2171513 no association with esophageal squamous cell carcinoma and esophagogastric junction adenocarcinoma

rs1844089, rs2705535, rs9288953, rs9288952 and rs16859633 [11-14]. Additionally we included in the study two tag dSNPs: rs1982809 and rs2705511 of *BTLA* gene, chosen with use of SNPinfo [15] on the basis of the criteria described in [16]

1. Fu, Z., et al., *Association of BTLA gene polymorphisms with the risk of malignant breast cancer in Chinese women of Heilongjiang Province.* Breast Cancer Res Treat, 2010. **120**(1): p. 195-202.

2. Cao, R., W. Tang, and S. Chen, *Association between BTLA polymorphisms and susceptibility to esophageal squamous cell carcinoma in the Chinese population.* J Clin Lab Anal, 2020. **34**(6): p. e23221.

3. Tang, W., et al., *Investigation of BTLA tagging variants with risk of esophagogastric junction adenocarcinoma.* Biosci Rep, 2019. **39**(12).

4. Ge, J., et al., *Association between co-inhibitory molecule gene tagging single nucleotide polymorphisms and the risk of colorectal cancer in Chinese.* J Cancer Res Clin Oncol, 2015. **141**(9): p. 1533-44.

5. Karabon, L., et al., *Intragenic Variations in BTLA Gene Influence mRNA Expression of BTLA Gene in Chronic Lymphocytic Leukemia Patients and Confer Susceptibility to Chronic Lymphocytic Leukemia.* Arch Immunol Ther Exp (Warsz), 2016. **64**(Suppl 1): p. 137-145.

6. Partyka, A., et al., *Association of 3' nearby gene BTLA polymorphisms with the risk of renal cell carcinoma in the Polish population.* Urol Oncol, 2016. **34**(9): p. 419 e13-9.

7. Khadhraoui, C., et al., *Association of BTLA rs1982809 polymorphism with lung cancer risk in Tunisian population.* Int J Immunogenet, 2020.
