# Supplementary material for: Immune Checkpoint Molecules—Inherited Variations as Markers for Cancer Risk
Source: Front Immunol. 2021 Jan 14;11:606721. doi: 10.3389/fimmu.2020.606721 (PMC7840570; doi:10.3389/fimmu.2020.606721)
Supplement: Supplementary file 5 [file Table_5.docx]

Supplementary Table 5. Summary of results concerning associations between *HAVCR2* polymorphisms and risk of different types of cancers.

| **Cancer** | ***HAVCR2* polymorphisms** | | | | | |
| --- | --- | --- | --- | --- | --- | --- |
|  | ***rs10053538***  ***(-1516 G>T*)** | ***rs891246256***  **(*-1541C>T*)** | ***rs4704853***  **(*-822C>T*)** | ***rs10515746***  **(*-574G>T*)** | ***rs1036199* (*+4259T>G*)** | ***rs4704846*** |
| **Overall cancer risk** | T+↑ [1]  T+↑ [2] | no data | T+↑ [2] | T+↑ [1]  T+↑ [2] | G+↑ [1]  G+↑ [2] | no data |
| **Breast cancer** | T+↑[3]  no association [4] | no data | no data | no association [4] | no association [3]  TG ↑ [4] | no association [3] |
| **Lung cancer** | no association [5] | no data | no data | no association [5] | TG ↑[5] | no data |
| **Gastrointestinal cancer** | T+↑ [1]  T+↑ [2] | no data | no data | T+↑ [1]  no association [2] | no data | no data |
| **Renal cell carcinoma** | no association [6] | no data | no data | GT ↑ [6] | TG ↑ [6] | no data |
| **Gastric cancer** | GT↑ [7] | no association [7] | CT ↑ [7] | GT ↑ [7] | no association [7] | no data |
| **Colorectal cancer** | no data | no data | TT ↑ [8] | no data | GG ↑ [8] | no data |
| **Pancreatic cancer** | no association [9] | no data | no data | no association [9] | TG ↑ [9] | no data |
| **Hematological malignances (NHL)** | no association [10] | no data | no data | GT ↑ [10] | TG ↑ [10] | no data |

1. Gao, X., et al., *Quantitative assessment of TIM-3 polymorphisms and cancer risk in Chinese Han population.* Oncotarget, 2016. **7**(24): p. 35768-35775.

2. Fang, H., et al., *Association between TIM-3 polymorphisms and cancer risk: a meta-analysis.* Ann Transl Med, 2019. **7**(20): p. 550.

3. Wang, Z., et al., *Polymorphisms in TIM-3 and breast cancer susceptibility in Chinese women: A case-control study.* Oncotarget, 2016. **7**(28): p. 43703-43712.

4. Cheng, S., et al., *T Cell Immunoglobulin- and Mucin-Domain-Containing Molecule 3 Gene Polymorphisms and Susceptibility to Invasive Breast Cancer.* Ann Clin Lab Sci, 2017. **47**(6): p. 668-675.

5. Bai, J., et al., *T-cell immunoglobulin- and mucin-domain-containing molecule 3 gene polymorphisms and prognosis of non-small-cell lung cancer.* Tumour Biol, 2013. **34**(2): p. 805-9.

6. Cai, C., et al., *T-cell immunoglobulin- and mucin-domain-containing molecule 3 gene polymorphisms and renal cell carcinoma.* DNA Cell Biol, 2012. **31**(7): p. 1285-9.

7. Cao, B., et al., *Genetic variations and haplotypes in TIM-3 gene and the risk of gastric cancer.* Cancer Immunol Immunother, 2010. **59**(12): p. 1851-7.

8. Zhang, P., et al., *Downregulated Tim-3 expression is responsible for the incidence and development of colorectal cancer.* Oncol Lett, 2018. **16**(1): p. 1059-1066.

9. Tong, D., et al., *T cell immunoglobulin- and mucin-domain-containing molecule 3 gene polymorphisms and susceptibility to pancreatic cancer.* Mol Biol Rep, 2012. **39**(11): p. 9941-6.

10. Song, H., et al., *T-cell immunoglobulin- and mucin-domain-containing molecule 3 genetic variants and HIV+ non-Hodgkin lymphomas.* Inflammation, 2013. **36**(4): p. 793-9.
